# Longitudinal CE-MRI-based Siamese network with machine learning to predict tumor response in HCC after DEB-TACE

**DOI:** 10.1186/s40644-025-00926-5

**Published:** 2025-08-19

**Authors:** Nan Wei, René Michael Mathy, De-Hua Chang, Philipp Mayer, Jakob Liermann, Christoph Springfeld, Michael T Dill, Thomas Longerich, Georg Lurje, Hans-Ulrich Kauczor, Mark O. Wielpütz, Osman Öcal

**Affiliations:** 1 Department of Diagnostic and Interventional Radiology, University Hospital of Heidelberg, Im Neuenheimer Feld 420, 69120 Heidelberg, Germany; 2 Liver Cancer Center Heidelberg, Im Neuenheimer Feld 460, 69120 Heidelberg, Germany; 3 National Center for Tumor Diseases (NCT), NCT Heidelberg, a partnership between DKFZ and University Hospital of Heidelberg, Heidelberg, Germany; 4 Institute of Radiology and Nuclear Medicine, Cantonal Hospital Lucerne, Spitalstrasse, Lucerne, CH-6000 Switzerland; 5 Department of Radiation Oncology, University Hospital of Heidelberg, Im Neuenheimer Feld 400, 69120 Heidelberg, Germany; 6 Department of Medical Oncology, University Hospital of Heidelberg, Im Neuenheimer Feld 460, 69120 Heidelberg, Germany; 7 Department of Gastroenterology, Hepatology, Infectious Diseases and Intoxication, University Hospital of Heidelberg, Im Neuenheimer Feld 410, 69120 Heidelberg, Germany; 8 German Cancer Research Center (DKFZ) Heidelberg, Experimental Hepatology, Inflammation and Cancer, Heidelberg, Germany; 9 Institute of Pathology, University Hospital of Heidelberg, Im Neuenheimer Feld 410, 69120 Heidelberg, Germany; 10 Department of General, Visceral and Transplantation Surgery, University Hospital of Heidelberg, Im Neuenheimer Feld 420, 69120 Heidelberg, Germany; 11 Department of Diagnostic Radiology and Neuroradiology, University Medicine Greifswald, Ferdinand-Sauerbruch-Strasse 1, 17475 Greifswald, Germany; 12 Clinic for Nuclear Medicine, University Medicine Greifswald, Ferdinand-Sauerbruch-Strasse 1, 17475 Greifswald, Germany

**Keywords:** Deep learning, Machine learning, Siamese network, HCC, Tumor response

## Abstract

**Background:**

Accurate prediction of tumor response after drug-eluting beads transarterial chemoembolization (DEB-TACE) remains challenging in hepatocellular carcinoma (HCC), given tumor heterogeneity and dynamic changes over time. Existing prediction models based on single timepoint imaging do not capture dynamic treatment-induced changes. This study aims to develop and validate a predictive model that integrates deep learning and machine learning algorithms on longitudinal contrast-enhanced MRI (CE-MRI) to predict treatment response in HCC patients undergoing DEB-TACE.

**Methods:**

This retrospective study included 202 HCC patients treated with DEB-TACE from 2004 to 2023, divided into a training cohort (*n* = 141) and validation cohort (*n* = 61). Radiomics and deep learning features were extracted from standardized longitudinal CE-MRI to capture dynamic tumor changes. Feature selection involved correlation analysis, minimum redundancy maximum relevance, and least absolute shrinkage and selection operator regression. The patients were categorized into two groups: the objective response group (*n* = 123, 60.9%; complete response = 35, 28.5%; partial response = 88, 71.5%) and the non-response group (*n* = 79, 39.1%; stable disease = 62, 78.5%; progressive disease = 17, 21.5%). Predictive models were constructed using radiomics, deep learning, and integrated features. The area under the receiver operating characteristic curve (AUC) was used to evaluate the performance of the models.

**Results:**

We retrospectively evaluated 202 patients (62.67 ± 9.25 years old) with HCC treated after DEB-TACE. A total of 7,182 radiomics features and 4,096 deep learning features were extracted from the longitudinal CE-MRI images. The integrated model was developed using 13 quantitative radiomics features and 4 deep learning features and demonstrated acceptable and robust performance with an receiver operating characteristic curve (AUC) of 0.941 (95%CI: 0.893–0.989) in the training cohort, and AUC of 0.925 (95%CI: 0.850–0.998) with accuracy of 86.9%, sensitivity of 83.7%, as well as specificity of 94.4% in the validation set.

**Conclusions:**

This study presents a predictive model based on longitudinal CE-MRI data to estimate tumor response to DEB-TACE in HCC patients. By capturing tumor dynamics and integrating radiomics features with deep learning features, the model has the potential to guide individualized treatment strategies and inform clinical decision-making regarding patient management.

**Supplementary Information:**

The online version contains supplementary material available at 10.1186/s40644-025-00926-5.

## Background

Hepatocellular carcinoma (HCC) is the eighth most common cancer and the third leading cause of cancer mortality worldwide [1]. Due to its asymptomatic onset, high heterogeneity, and aggressive progression, about 70% of patients are diagnosed at advanced stages [2–4]. Given this context, drug-eluting beads transarterial chemoembolization (DEB-TACE) emerges as one of the primary therapeutic approaches for intermediate to advanced HCC [5, 6].

Despite its efficacy, the effectiveness remains variable due to the heterogeneity of HCC, requiring multiple DEB-TACE in the same nodule to achieve adequate tumor control [7]. Radiologic imaging, particularly CE-MRI or CE-CT, offers a non-invasive and reproducible means of assessing tumor response, evaluated by the modified Response Evaluation Criteria in Solid Tumors (mRECIST) [8]. The mRECIST classification stratifies tumor responses into complete response (CR), partial response (PR), stable disease (SD), and progressive disease (PD) categories [8]. While some patients respond well to DEB-TACE and exhibit significant tumor reduction after a single procedure, others may experience limited treatment effects, leading to resistance and tumor progression [9]. This stratification is essential for guiding personalized treatment strategies, but challenging to predict individual patient responses, leading to treatment delay or suboptimal therapy choices for non-responders.

In response to these challenges, recent advances in artificial intelligence introduced deep learning (DL) [10–12] and machine learning (ML) [13–26] methods as promising tools for predicting HCC treatment outcomes. However, the tumor is a dynamic biological system influenced by vascular and stem cell factors, which can change over time, meaning that its phenotype may not be fully captured at a single timepoint [27]. Previous studies focus on utilizing CE-MRI [20–23, 25, 26, 28] or CE-CT [10–19, 24, 29] from pre-conventional-TACE to construct predictive models for tumor response, using the tumor response results from follow-up images taken 4–6 weeks post-intervention as the prediction target. These approaches, which analyze images obtained at a single time point from patients, failed to predict the long-term changes after treatment effectively and were not able to incorporate information from longitudinal imaging of treatment induced changes [30]. Especially, DEB-TACE can lead to distinct changes in the vascularity of the tumor and perifocal hepatic tissue [31]. Recently, DL and ML have been applied to analyze longitudinal MRI, CT, X-ray or ultrasound images to predict disease risk or progression [27, 30, 32–39]. However, this approach has not yet been explored in HCC. Thus, we aim to develop and validate a predictive model to assess treatment response in HCC patients undergoing DEB-TACE.

## Methods

### Study design and participants

This single-center, retrospective study was conducted at a large center for interventional oncology. The study protocol was approved by the Institutional Ethics Committee of Heidelberg University Hospital (No. S-346/2024) and complied with the Declaration of Helsinki. All patient information was anonymized before analysis. All patients diagnosed with HCC who underwent DEB-TACE as their initial treatment from January 2004 to December 2023 were evaluated. Clinical and laboratory data were extracted from electronic medical records.

**Fig. 1:**
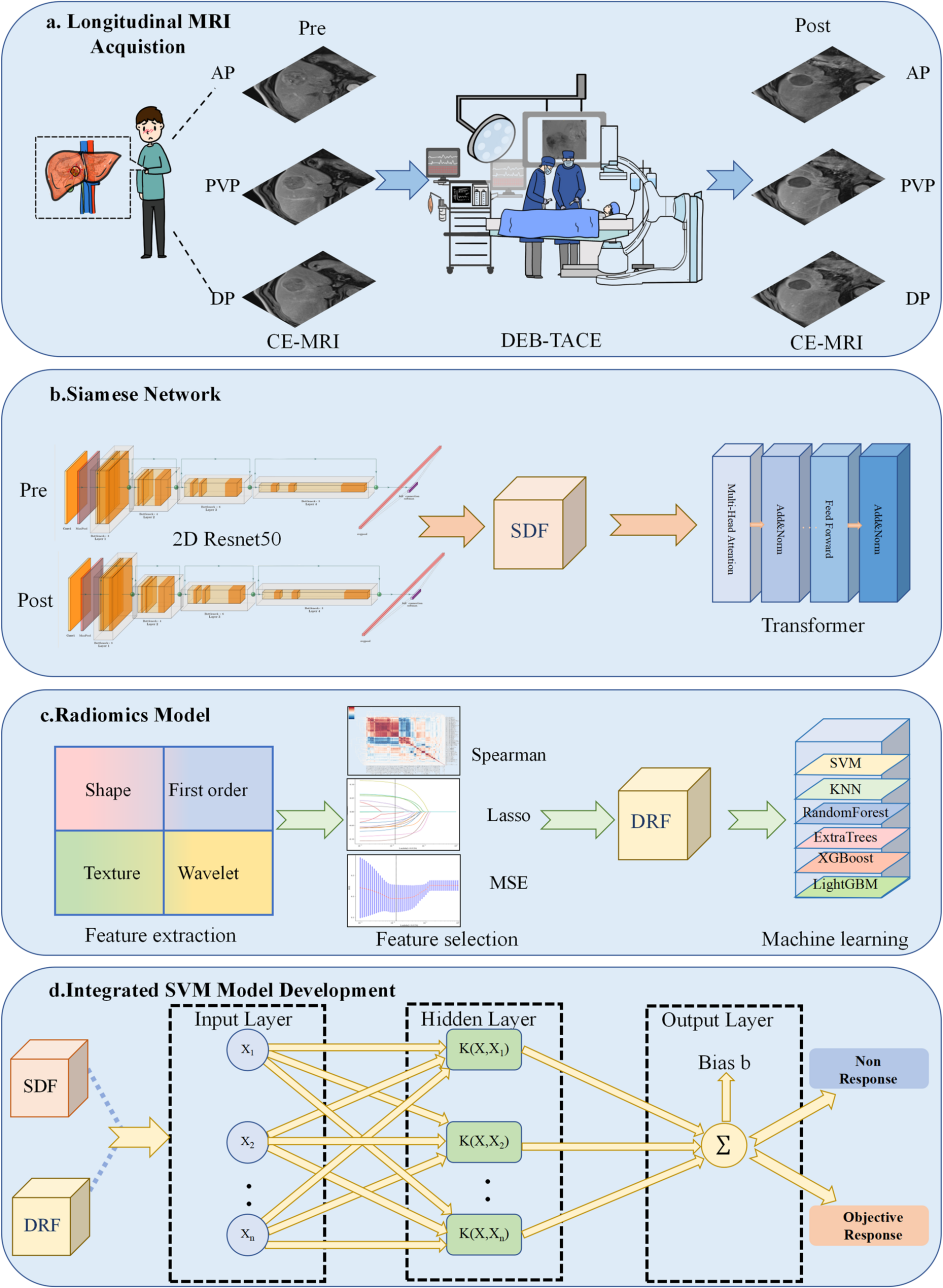
The study design and workflow of longitudinal CE-MRI-based Siamese network with machine learning to predict tumor response in HCC after DEB-TACE. SDF Siamese delta features; Lasso Least absolute shrinkage and selection operator; MSE Minimum mean squared error; DRF Delta radiomic features; SVM, support vector machine; KNN, nearest neighbors; XGBoost, extreme gradient boosting; LightGBM, light gradient boosting machine;

For a detailed overview of the inclusion and exclusion process, please refer to Supplementary Material S1. After applying all these criteria, a total of 202 patients remained eligible for the study. Patients were randomly assigned to either the training or testing cohort in a 7:3 ratio, used for training and validating the DL and ML models. The study design and workflow are illustrated in Fig. 1.

### Treatment strategies and tumor response assessment

All DEB-TACE were performed by experienced interventional radiologists with over ten years of clinical expertise. Techinical details are provided in Supplementary Material S2. Patients underwent regular follow-up with CE-MRI at 1–2 months post-treatment and every 3–4 months thereafter. Clinical evaluations and alpha-fetoprotein (AFP) tests are conducted every three months. Tumor response was evaluated based on T1-weighted imaging (T1WI) in the arterial phase (AP) before and after DEB-TACE, following the mRECIST1.1 as detailed in Supplementary Material S3. For this study, patients were further grouped into two categories based on the third CE-MRI assessment, which was conducted within 3 months after the first post-DEB-TACE follow-up: the objective response (OR) group, which included those with CR or PR, and the non-response (NR) group, comprising patients with SD or PD. Two experienced abdominal radiologists, each with 5 years of experience, independently assessed tumor response to ensure consistency, and in cases of disagreement, a third physician with 8 years of experience was consulted to achieve a consensus.

### MRI acquisition and post-processing

All MRI scans were performed on 1.5 or 3.0 Tesla MR scanner (Siemens Healthineers, Erlangen, Germany), first imaging being within two months (median: 25 days; IQR: 10–37 days) before the DEB-TACE procedure, second within two months after DEB-TACE (median: 29 days; IQR: 28–35 days), and the third, performed for tumor response evaluation purposes, was acquired three months after the second MRI (median: 98 days; IQR: 94–112 days). The MRI protocol included T1WI in AP, portal venous phase (PVP), and delayed phase (DP) after gadolinium-based contrast administration, as detailed in Supplementary Material S4. Variations in imaging protocols across different scanners were addressed by applying a standardized preprocessing pipeline, including voxel resampling to a uniform spacing of 1.0 × 1.0 × 1.0 mm3, z-score intensity normalization, and N4 bias field correction, to minimize scanner- and time-related variability. Data augmentation techniques were additionally used during network and machine learning training. Detailed post-processing methods are provided in Supplementary Material S5.

### Tumor segmentation and radiomics analysis

Two abdominal radiologists (each with 5 years’ experience) manually segmented tumor regions of interest (ROIs) on AP, PVP, and DP images using 3D Slicer Software (v5.6.2), avoiding vessels and bile ducts. In cases of disagreement, a senior radiologist with more than 10 years of expertise reviewed and finalized the segmentation. Manual delineation of the tumor lesions for each patient was performed layer by layer on the images of each sequence, outlining the ROI. In total, 7182 features were derived from the tumor ROI across the various MRI sequences. Further details and explanations of these features can be found in Supplementary Material S6.

### Deep learning network

The DL model was designed using a dual 2D ResNet50 structure with a Transformer for feature integration. Each ResNet50 extracted deep learning features (DLF) from pre- and post-DEB-TACE CE-MRI images, allowing for comparative analysis over time. The fully connected layers of the ResNet50 networks extracted deep learning features (DLF)from each patient’s pre- and post-treatment images. To capture dynamic treatment effects, the Siamese network calculated the SDF by comparing DLF from pre- and post-treatment images. These DLF were subsequently integrated within a Transformer network, enhancing the model’s ability to recognize longitudinal changes that are critical for treatment response prediction. Comprehensive details regarding the network architecture, loss function and network training details are provided in the Supplementary Material S7.

### Feature selection

To assess feature reliability, 20 cases were randomly selected and independently segmented by two radiologists. Features with intraclass correlation coefficients (ICC) < 0.75 were excluded. For the remaining dataset, segmentations from one radiologist were used. To identify key features associated with tumor response, we applied univariate tests (Mann-Whitney U and t-test), Spearman correlation for redundancy, the minimum redundancy maximum relevance (MRMR) algorithm for relevance filtering, and the least absolute shrinkage and selection operator (LASSO) logistic regression for final feature selection. For DLF extracted using 2D ResNet50, principal component analysis (PCA) was applied for dimensionality reduction before conducting the aforementioned feature selection methods. The final selected features were then used to build ML models, with LASSO regression modelling performed using the Python scikit-learn package (version 1.5.2). Detailed information on the feature selection process is provided in Supplementary Material S8.

### Development and assessment of models

Model development and validation were performed using scikit-learn (v1.5.2) in Python 3.13.0. The validation cohort was utilized for the final assessment of the constructed models. We incorporated the selected DRF and/or SDF to develop a total of 9 models, categorized into pre-, post-, and delta-models. This modelling process was framed as a supervised learning task, guided by the labels corresponding to OR and NR. A variety of 6 robust classification algorithms were employed, namely Support Vector Machine (SVM), K Nearest Neighbors, Random Forest, eXtreme Gradient Boosting, Light Gradient Boosting Machine, and Extratrees. Evaluation metrics included the area under the curve (AUC) with a 95% confidence interval (CI), specificity, sensitivity, accuracy, positive predictive value (PPV), negative predictive value (NPV), precision, recall, and F1 score. Comprehensive details regarding the ML workflow and methodologies can be found in Supplementary Material S9.

### SVM model explainability

To enhance interpretability, we used SHapley Additive exPlanations (SHAP) with the kernel explainer to interpret the SVM model. SHAP computes feature contributions to each prediction, allowing both global and individual interpretation. Key visualizations, such as SHAP beeswarm, waterfall, and force plots, helped clarify the role of each feature in the model’s decision-making process. Detailed SHAP analysis and visualizations are provided in Supplementary Material S10.

### Statistical analysis

The baseline characteristics were analyzed using R 3.4.2 and Python 3.13.0. Continuous variables were presented as mean ± standard deviation, while categorical variables were represented as frequencies and percentages. The distribution of continuous variables was assessed using the Shapiro-Wilk test to determine normality. Variance homogeneity was evaluated through Levene’s test. For inter-group comparisons, the Mann-Whitney U test or independent samples t-test was employed based on data distribution. Differences in categorical variables were analyzed using either the Chi-squared test or Fisher’s exact test as appropriate. *p* value < 0.05 was considered indicative of statistical significance. The DeLong test was utilized to compare the AUC of different models.

## Results

### Baseline characteristics of patients

A total of 202 patients with HCC were included, comprising 169 (83.7%) males and 33 (16.4%) females, with a median age of 62.7 ± 9.3 years (range: 34–89 years). The training cohort (*n* = 141, 69.8%) included 114 (80.9%) males and 27 (19.1%) females, with a mean age of 63.3 ± 9.1 years, while the validation cohort (*n* = 61, 30.2%) included 55 (90.2%) males and 6 (9.8%) females, with a mean age of 61.3 ± 9.5 years. Based on the third CE-MRI assessment, 35 patients (17.3%) achieved CR, 88 (43.6%) had PR, 62 (30.7%) demonstrated SD, and 17 (8.4%) exhibited PD according to the mRECIST criteria. For pre-treatment scans 186 of 202 (92.1%) were obtained at 1.5T, as well as 189 (93.6%) of post-treatment scans. Field strength remained consistent between pre- and post-treatment imaging in 185 patients (91.6%), while 17 patients (8.4%) underwent imaging with different field strengths (1.5T to 3.0T = 7, 3.5%; 3.0T to 1.5T = 10, 4.9%). As outlined in Table 1, baseline characteristics for both cohorts showed no statistically significant differences in demographics, clinical characteristics, or radiological features. In addition, in the validation cohort, post-interventional AFP levels differed significantly between OR and NR groups (*p* < 0.05), as we expected.

### Feature extraction and selection

**Fig. 2:**
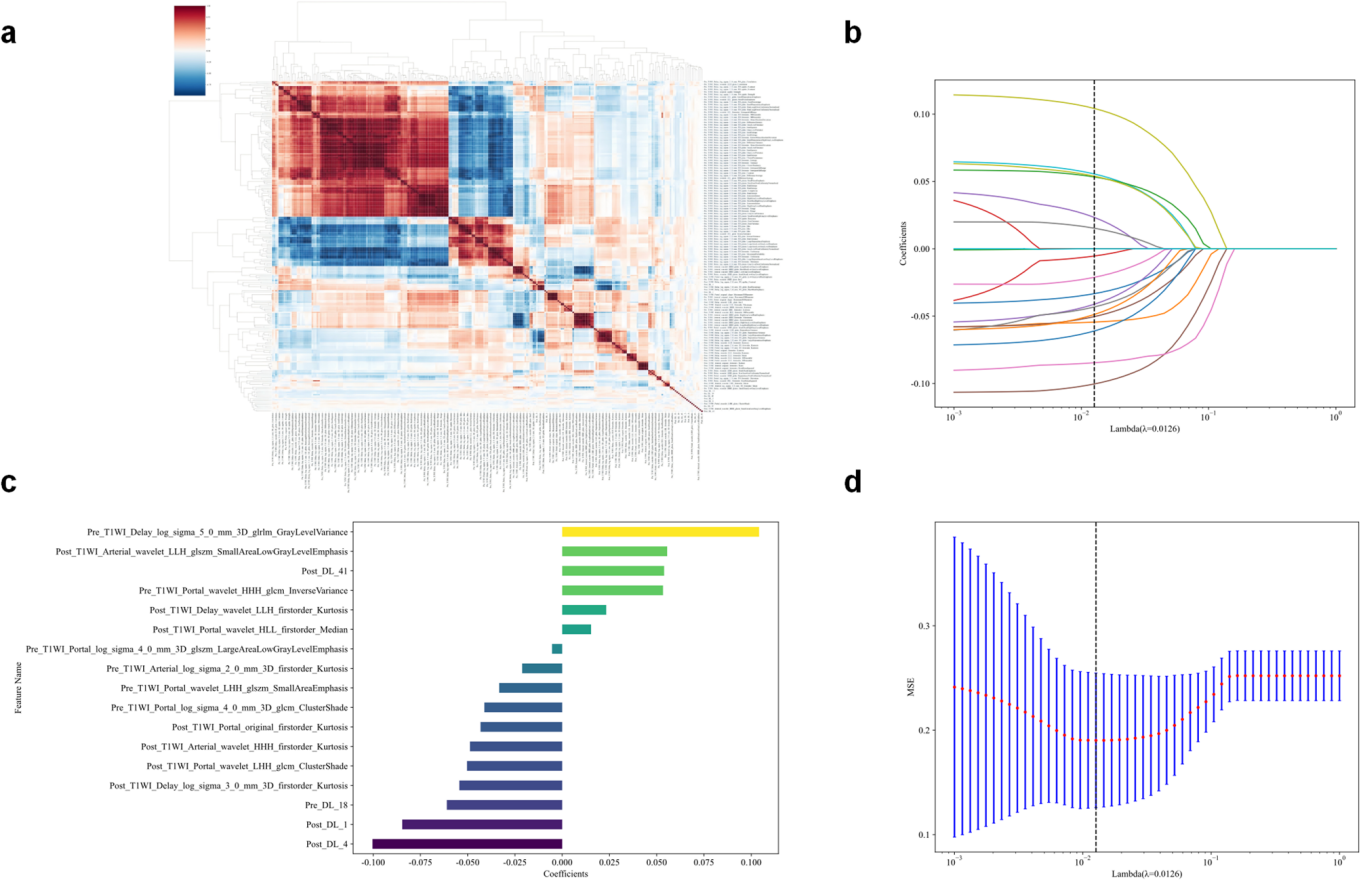
Radiomic feature selection using the Maximum relevance minimum redundancy (MRMR) and the least absolute shrinkage and selection operator (LASSO). (**a**) A clustering heatmap of Spearman rank correlation selected features deriving from longitudinal CE-MRI images. The resulting heatmap is visualized using a color bar of the z-score, a higher z-score with a more red display and a lower z-score with a more blue display. (**b**) Selection of the tuning parameter (λ) in the LASSO model using five-fold cross-validation via minimum mean squared error (MSE). Dotted vertical lines drawn at the optimal values of 0.0126 were selected; (**c**) The histogram exhibits radiomics features contributed to the constructed radiomics model. The y-axis represents radiomics features, with their coefficients in the multivariate logistic regression analysis plotted on the x-axis. (**d**) LASSO coefficient profiles of the radiomic features. The dotted vertical line was plotted at the optimal λ, resulting in 17 features with nonzero coefficients

A total of 7,182 radiomic features and 4,096 DLF were extracted. To ensure reproducibility, of handcrafted radiomics features, 20(10.0%) patients were randomly selected for interobserver agreement analysis, and 1,416 features with an ICC < 0.75 were excluded. For DLF, PCA reduced the feature set to 128 components. The Mann-Whitney U test and t test identified 261 features significantly associated with tumor response outcomes, with Spearman correlation coefficients illustrating inter-feature associations. Applying the MRMR algorithm further refined the selection of 20 key features. To refine the selection further, LASSO feature selection was implemented, yielding an optimal subset of 17 features, as shown in Fig. 2. The final feature sets for the DL_Rad_Delta model include a combination of handcrafted radiomics features and deep learning features. A detailed description of the selected features for all models is available in Supplementary Material S11.

### Development and performance of models

**Fig. 3:**
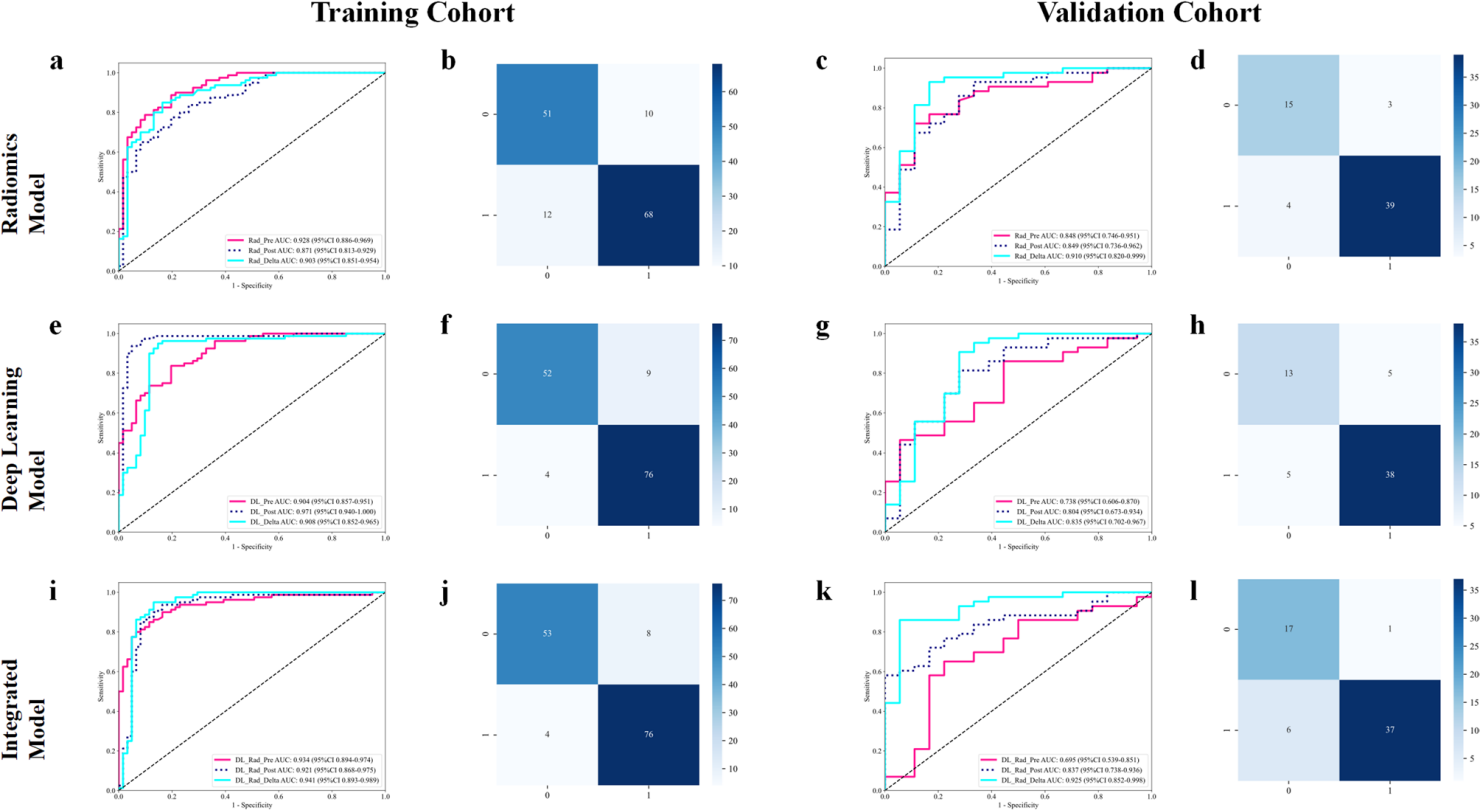
The receiver operating characteristic (ROC), confusion matrix of the different models in the training and validation cohorts (**a-i**). The ROC curves of the radiomics model, deep learning model, and integrated model in the training (**a, e, i**) and validation (**c, g, k**) cohorts. The confusion matrix of the radiomics model, deep learning model, and integrated model in the training (**b, f, j**) and validation (**d, h, l**) cohorts

Six ML algorithms were tested across all radiomic feature sets, with the SVM Rad_Delta model demonstrating the best performance among ML-only models. Specifically, in the training cohort, the SVM Rad_Delta model achieved an AUC of 0.903 (95% CI: 0.851–0.954), and in the validation cohort, an AUC of 0.910 (95% CI: 0.820–0.999). For DL-only models, the DL_Delta model achieved an AUC of 0.908 (95% CI: 0.852–0.965) in the training cohort and 0.835 (95% CI: 0.702–0.967) in the validation cohort. Among integrated models, the SVM DL_Rad_Delta model, which combines both DRF and SDF, achieved the highest performance, with an AUC of 0.941 (95% CI: 0.893–0.989) in the training cohort and 0.925 (95% CI: 0.852–0.998) in the validation cohort, as shown in Table 2; Fig. 3. A comparative analysis of the best SVM models from each approach (Rad_Delta, DL_Delta, and DL_Rad_Delta) using the DeLong test revealed no statistically significant differences in performance between models (*p* > 0.05) in both training and validation cohorts. However, in the training cohort, the integrated DL_Rad_Delta model achieved the highest values in accuracy, specificity, AUC, PPV, NPV, precision, and F1 score, outperforming the DL_Delta and Rad_Delta models. For sensitivity and recall, both the integrated model and the DL_Delta model showed similar performance, both surpassing the Rad_Delta model. In the validation cohort, the integrated model consistently outperformed both DL_Delta and Rad_Delta across these metrics, including specificity, AUC, PPV, and precision. These findings indicate that the DL_Rad_Delta model, integrating both DRF and SDF, offers the most robust and accurate predictions of tumor response, establishing it as the optimal model for potential clinical application. The DeLong test results, and performance metrics of other models can be found in Supplementary Material S12 and S13.

### Explainability of the model

**Fig. 4:**
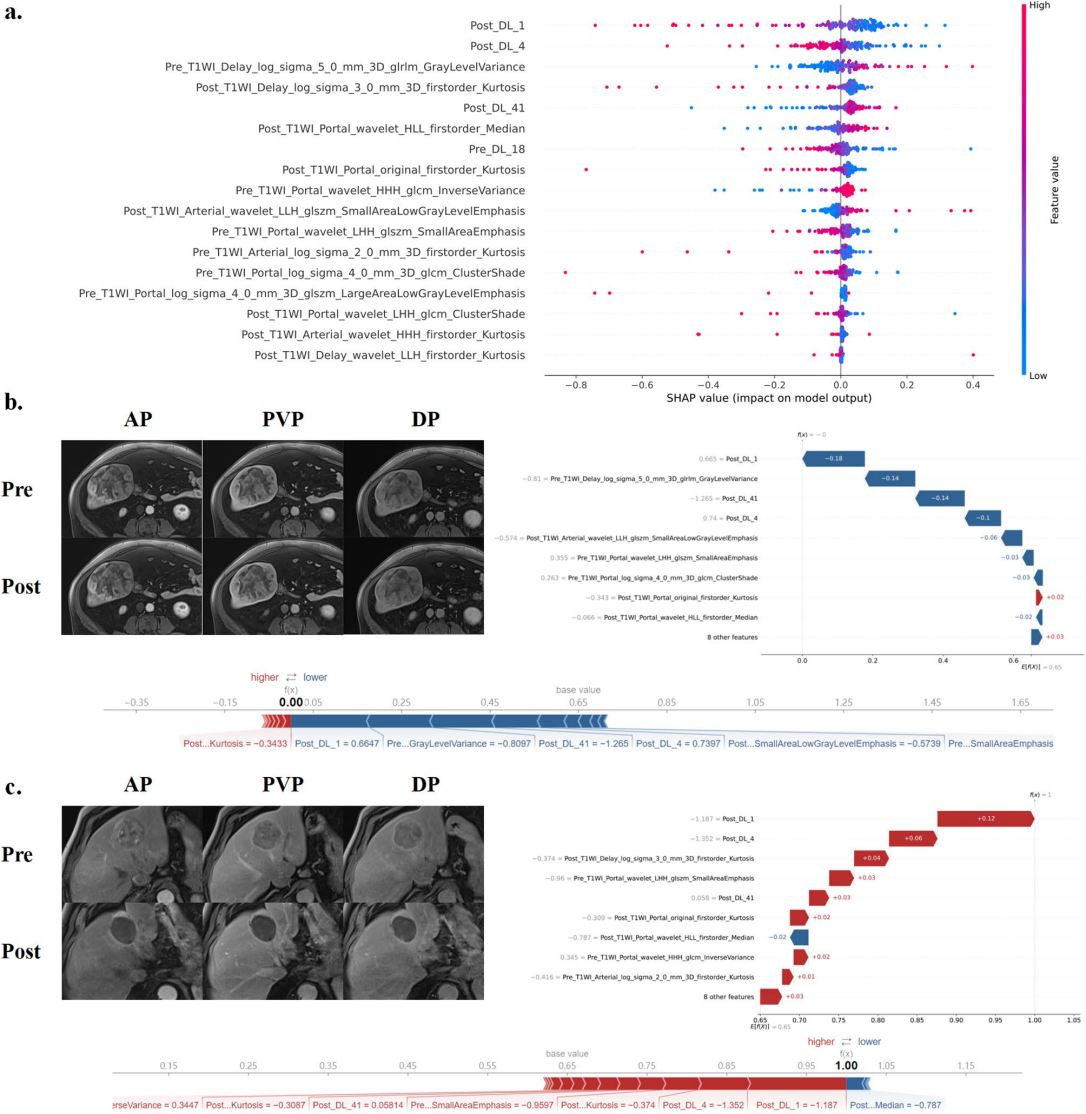
Integrated delta SVM model explainability. (**a**) SHAP beeswarm plots of the model. The plot illustrated the feature relevance and combined feature attributions to the model’s predictive performance. CE-MRI images show the tumor in the arterial phase (AP), portal venous phase (PVP) and delayed phase (DP) before and after DEB-TACE from a 69-year-old man with a 119 mm lesion in the left lobe of the liver (**b**) and a 72-year-old man with a 35 mm lesion in the right lobe of the liver (**c**). The SHAP waterfall plot summarizes the contribution of individual imaging features to the model’s prediction for this patient, with the x-axis representing the model output and features ranked by their impact. Features with negative SHAP values (blue) decrease the prediction score, while features with positive SHAP values (red) increase it. The SHAP force plot further illustrates the cumulative effect of all features, starting from the baseline prediction *(E[f(x)] = 0.65)* to the final model output

In Fig. 4a, the SHAP analysis identified Post_DL_1 as the most influential feature in distinguishing OR from NR. For one patient in Fig. 4b, the computed SHAP value (*f(x) =* 0.00) fell below the base value (0.65), indicating an NR classification driven predominantly by a negative contribution from Post_DL_1. In contrast, for another patient, as shown in Fig. 4c, the SHAP value (*f(x) =* 1.00) exceeded the base value, suggesting an OR classification. In this case, Post_DL_1 provided a substantial positive contribution. These findings demonstrate that variations in Post_DL_1 significantly influence model predictions, underscoring its role as a key predictive indicator.

## Discussion

This study is the first to utilize longitudinal CE-MRI data combined with multimodal DL and ML techniques to develop a model for predicting long-term tumor response in HCC patients undergoing DEB-TACE. Our results demonstrate that the model exhibits good performance in distinguishing NR and OR patients. Accurate tumor response assessment is essential for optimizing treatment strategies, improving outcomes, predicting prognosis, and minimizing adverse effects [40]. Recently, multimodal ML and DL models leveraging MRI have shown promise in tumor response evaluation following TACE [12–14]. However, there is currently a lack of studies utilizing longitudinal CE-MRI, coupled with multimodal DL and ML, to predict tumor response in HCC with DEB-TACE. To address this, we developed a model based on longitudinal CE-MRI, aiming to enhance predictive accuracy. In this study, we also developed several other models, including ML models constructed using only radiomic features (preoperative, postoperative, and Delta), DL models constructed using only DLF (preoperative, postoperative, and Delta: Transformer-based fusion), as well as models combining radiomic features and DLF using only preoperative data and only postoperative data.

We also evaluated the predictive performance of the Delta model and compared it to pre-treatment and post-treatment models using DeLong tests. In the training cohort, the Delta model did not demonstrate statistically significant differences compared to Pre and Post models in radiomics (*p* = 0.269 and 0.291), DL (*p* = 0.909 and 0.059), or integrated models (*p* = 0.820 and 0.275). In the validation cohort, the Delta model also showed no statistically significant differences compared to Pre and Post models in radiomics (*p* = 0.372 and 0.352) and DL (*p* = 0.299 and 0.698). However, in the integrated models, the Delta model achieved statistical significance when compared to the Pre model (*p* = 0.009) and showed near-significance when compared to the Post model (*p* = 0.063), as shown in Supplementary Fig. 3 and Fig. 4. Although some differences did not reach statistical significance, the Delta model exhibited the best overall performance in both the training and validation cohorts. For instance, the AUCs of the integrated Delta model reached 0.941 and 0.925 in the training and validation cohorts, respectively, emphasizing its capacity to encapsulate dynamic tumor changes induced by treatment.

The performance of the Delta model suggests that capturing biological changes in tumors caused by DEB-TACE treatment can improve prediction accuracy. These changes may include tumor necrosis, reduced vascularity, and altered tumor heterogeneity, data from a single time point, such as pre-treatment or post-treatment imaging alone cannot fully reflect. By quantifying the differences in features before and after treatment, the Delta model can provide more sensitive and specific predictions, enabling clinicians to adjust treatment strategies promptly and avoid the adverse effects of ineffective therapies. However, the lack of statistically significant differences in the DeLong tests may be attributed to the relatively small sample size, which limits the statistical power to detect differences between models. Therefore, future studies with larger, multi-center cohorts are needed to validate these findings and determine whether the observed trends are statistically significant. Although delta features capture longitudinal changes within each patient and help identify treatment response, imaging heterogeneity remains a concern. This issue is particularly relevant because our dataset spans a long period, during which different MRI scanners and acquisition protocols were used, potentially introducing temporal variability. In addition, differences in magnetic field strength between 1.5T and 3.0T scanners can affect image contrast, signal-to-noise ratio, and spatial resolution—factors that influence radiomic features’ stability [41]. Several studies have reported that many radiomic features, especially texture-based ones, vary significantly between 1.5T and 3.0T scans, even when the same acquisition protocols and MRI scanners are applied [42, 43]. Our preprocessing steps- including voxel resampling, z-score normalization, and N4 bias field correction- were applied to reduce such variability. However, residual effects cannot be entirely excluded and should be acknowledged when evaluating model robustness.

This research study shows several benefits over previous research. First, our integrated ML and DL model, which incorporates MRI data, offers improved predictive accuracy compared to approaches relying solely on clinical features, radiomics, or DL in isolation. Second, we employed longitudinal CE-MRI data, capturing multiple time points before and after DEB-TACE, to reflect dynamic changes in tumor size, enhancement, and vascularization. This method gives a better understanding of tumor progression, unlike earlier studies that used only pre-treatment imaging and missed post-treatment dynamics. Third, we evaluated different modeling approaches, including preoperative, postoperative, and combined strategies. The “Delta” model outperformed others, with AUC values of 0.910, 0.835, 0.925 and 0.903, 0.908, 0.941 across validation and training sets, surpassing the “only-pre” and “only-post” models. These results emphasize the necessity of capturing tumor changes during DEB-TACE for accurate response prediction. Additionally, while SVM-based models are common in clinical studies, their lack of interpretability limits their practical use [44]. To address this, we incorporated SHAP to provide clinicians with a more comprehensive insight into the SVM model’s predictions. SHAP beeswarm, waterfall, and force plots offer insight into the impact of individual features on model output, providing a more intuitive interpretation compared to traditional feature importance plots [45, 46]. Despite the promising findings regarding the model’s predictive capabilities, several limitations must be addressed. First, as a single-center, retrospective study, there is a potential for selection bias, and the small sample size requires larger prospective clinical trials to validate the model’s generalizability. Second, only internal validation was conducted, which limits the understanding of the model’s robustness. Testing with external datasets is important to ensure the model’s applicability across different populations. Additionally, variations in MRI acquisition parameters- such as magnetic field strength and protocol differences across the extended data collection period- may have introduced imaging heterogeneity. Although standardized preprocessing was applied to reduce such variability, residual effects on radiomic features and model generalizability cannot be fully excluded. These variations may impact the reproducibility and robustness of the model, and should be further addressed in future multi-center prospective studies involving harmonized imaging protocols. Furthermore, this study relied solely on CE-MRI data, and integrating other clinical and laboratory variables could further improve prediction performance. Finally, the ROI was manually delineated by radiologists, a time-consuming process prone to variability. Developing automated, reliable liver tumor segmentation tools is an essential subsequent phase.

**Table 1:** Baseline characteristics of patients in the training and validation cohort

Baseline Characteristic	Total (n=202)	Training cohort (n=141)				Validation cohort (n=61)				*p* value
		Total	OR group (n=80)	NR group (n=61)	*p* value	Total	OR group (n=43)	NR group (n=18)	*p* value	
Age (years, mean ± SD)^*^	62.67 ± 9.25	63.27 ± 9.11	62.85 ± 9.16	63.82 ± 9.09	0.533	61.30 ± 9.52	61.74 ± 10.04	60.22 ± 8.29	0.573	0.164
Gender (n,[%])					0.468				0.799	0.100
Male	169 (83.66)	114 (80.85)	63 (78.75)	51 (83.61)		55 (90.16)	38 (88.37)	17 (94.44)		
Female	33 (16.34)	27 (19.15)	17 (21.25)	10 (16.39)		6 (9.84)	5 (11.63)	1 (5.56)		
History of hepatitis B or C (n, [%])					0.245				0.934	0.640
Positive	91 (45.05)	61 (43.26)	42 (52.50)	38 (62.30)		30 (50.82)	21 (48.84)	9 (50.00)		
Negative	111 (54.95)	80 (56.74)	38 (47.50)	23 (37.70)		31 (49.18)	22 (51.16)	9 (50.00)		
Child-Pugh class (n,[%])					0.860				0.334	0.381
A	145 (71.78)	105 (74.47)	58 (72.50)	47 (77.05)		40 (65.57)	26 (60.47)	14 (77.78)		
B	47 (23.27)	29 (20.57)	18 (22.50)	11 (18.03)		18 (29.51)	15 (34.88)	3 (16.67)		
C	10 (4.95)	7 (4.96)	4 (5.00)	3 (4.92)		3 (4.92)	2 (2.46)	1 (5.56)		
BCLC stage (n, [%])					0.346				0.498	0.399
A	98 (48.51)	64 (45.39)	40 (50.00)	24 (39.34)		34 (55.74)	26 (60.47)	8 (44.44)		
B	80 (39.61)	59 (41.84)	32 (40.00)	27 (44.26)		21 (34.43)	13 (30.23)	8 (44.44)		
C	24 (11.88)	18 (12.77)	8 (10.00)	10 (16.39)		6 (9.83)	4 (9.3)	2 (11.12)		
Diabetes (n, [%])					0.491				0.480	0.803
Absent	135 (66.83)	95 (67.38)	52 (65.00)	43 (70.49)		40 (65.57)	27 (62.79)	13 (72.22)		
Present	67 (33.17)	46 (32.62)	28 (35.00)	18 (29.51)		21 (34.43)	16 (37.21)	5 (27.78)		
Ascites (n, [%])					0.361				0.811	0.248
Absent	150 (74.26)	108 (76.6)	59 (73.75)	49 (80.33)		42 (68.85)	30 (69.77)	12 (66.67)		
Present	52 (25.74)	33 (23.4)	21 (26.25)	12 (19.67)		19 (31.15)	13 (30.23)	6 (33.33)		
Underlying liver disease (n, [%])					0.877				0.241	1.000
NAFLD	5 (2.48)	4 (2.83)	3 (3.75)	1 (1.64)		1 (1.64)	1 (2.33)	0 (0.00)		
Alcoholic liver disease	62 (30.69)	43 (30.50)	23 (28.75)	20 (32.79)		19 (31.15)	16 (37.20)	3 (16.67)		
Other	4 (1.98)	3 (2.13)	2 (2.50)	1 (1.64)		1 (1.64)	1 (2.33)	0 (0.00)		
Absent	131 (64.85)	91 (64.54)	52 (65.00)	39 (63.93)		40 (65.57)	25 (58.14)	15 (83.33)		
Pre DEB-TACE AFP (ng/mL)^†^					0.584				0.498	0.837
≤200	164 (81.19)	115 (81.56)	64 (80.00)	51 (83.61)		49 (80.33)	36 (83.72)	13 (72.22)		
>200	38 (18.81)	26 (18.44)	16 (20.00)	10 (16.39)		12 (19.67)	7 (16.28)	5 (27.78)		
Post DEB-TACE AFP (ng/mL)^†^					0.405				***0.010***	0.864
≤200	181 (89.60)	126 (89.36)	73 (91.25)	53 (86.89)		55 (90.16)	42 (97.67)	13 (72.22)		
>200	21 (10.40)	15 (10.64)	7 (8.75)	8 (13.11)		6 (9.84)	1 (2.33)	5 (27.78)		
AST (U/L)^†^	59.00 (41.00, 90.00)	58.50 (41.00, 90.00)	51.00 (38.00, 95.25)	67.50 (48.75, 86.00)	0.125	59.00 (45.00, 90.00)	63.00 (32.50, 91.50)	50.50 (45.00, 82.75)	0.613	0.545
ALT (U/L)^†^	46.00(28.75, 75.00)	44.00 (26, 74.00)	39.50 (25.00, 71.75)	50.00 (30.50, 74.50)	0.514	49.00 (34.00, 76.00)	47.00 (32.50, 76.00)	62.00 (37.75, 75.50)	0.342	0.292
GGT (U/L)^†^	139.00(74.00, 240.00)	142.00 (71.00, 253.00)	140.00 (67.00, 211.00)	158.00 (85.75, 301.75)	0.155	133.00 (89.00, 217.00)	133.00 (87.00, 198.00)	129.00 (94.00, 241.00)	0.975	0.601
Tbil (mg/dL)^†^	1.10 (0.80, 1.80)	1.10 (0.80, 1.80)	1.10 (0.80, 1.80)	1.20 (0.70, 1.75)	0.886	1.20 (0.80, 2.00)	1.30 (0.80, 2.20)	1.05 (0.72, 1.48)	0.241	0.428
ALB (g/L)^†^	37.40 (33.20, 41.90)	37.90 (32.95, 41.48)	38.20 (32.70, 41.40)	37.90 (33.20, 41.95)	0.966	36.50 (33.60, 41.88)	35.70 (31.53, 40.38)	38.75 (35.35, 42.53)	0.103	0.704
PLT (×10^9^/L)^†^	111.00 (72.00, 159.50)	117.00 (76.75, 153.00)	115.5 (75.00, 146.00)	119.50 (77.00, 175.00)	0.270	99.00 (69.75, 166.25)	100.50 (71.25, 163.00)	83.5 (65.5, 166.25)	0.723	0.369
INR ^†^	1.12 (1.05, 1.22)	1.11 (1.04, 1.20)	1.10 (1.04, 1.20)	1.12 (1.04, 1.21)	0.673	1.14 (1.08, 1.27)	1.13 (1.07, 1.27)	1.15 (1.08, 1.20)	0.975	0.237
PT (s)^†^	26.90 (25.50, 29.37)	26.90 (25.57, 29.37)	26.90 (25.45, 29.05)	27.70 (26.00, 29.70)	0.346	26.80 (25.45, 29.35)	26.80 (25.60, 29.28)	26.80 (25.40, 29.40)	0.955	0.750
ΔAFP (ng/mL)^†^	1.30 (-1.00, 34.50)	2.00 (-0.65, 35.40)	1.40 (-0.4,0 85.40)	2.65 (-1.07, 15.55)	0.269	0.20 (-2.00, 23.00)	0.20 (-0.93, 17.02)	0.80 (-29.70,42.15)	0.570	0.330
Tumor size (mm)^†^	35.00 (24.00, 50.00)	36.00 (24.00, 50.00)	37.50 (24.75, 50.00)	34.00 (23.00, 47.00)	0.730	35.00 (26.00, 55.00)	31.00 (23.50, 49.00)	42.50 (27.25, 73.75)	0.148	0.551

AFP, alpha-fetoprotein; ALB, albumin; ALT, alanine aminotransferase; AST, aspartate aminotransferase; BCLC, Barcelona Clinic Liver Cancer; GGT, γ-glutamyltranspeptadas; NAFLD, nonalcoholic fatty liver disease; NR, non-response; OR, objective response; PLT, platelet count; PT, prothrombin time; SD, standard deviation; DEB-TACE, drug-eluting beads transarterial chemoembolization; Tbil, total bilirubin; ΔAFP, Pre DEB-TACE AFP - Post DEB-TACE AFP

^*^ Data are means ± SDs, with ranges in parentheses

^†^ Data are medians, with IQRs in parentheses

**Table 2:** Discrimination performance of predictive models in the training and validation cohorts

Cohorts	Model Name	AUC	95% CI	Sensitivity	Specificity	PPV	NPV	Precision	Recall	Accuracy	F1
Training	Rad_Pre	0.928	0.8861–0.9692	0.875	0.803	0.854	0.831	0.854	0.875	0.844	0.864
	Rad_Post	0.871	0.8130–0.9292	0.762	0.803	0.836	0.721	0.836	0.762	0.780	0.797
	**Rad_Delta**	**0.903**	**0.8510–0.9543**	**0.837**	**0.836**	**0.870**	**0.797**	**0.870**	**0.837**	**0.837**	**0.854**
Validation	Rad_Pre	0.848	0.7456–0.9508	0.698	0.889	0.937	0.552	0.937	0.698	0.754	0.800
	Rad_Post	0.849	0.7357–0.9620	0.907	0.667	0.867	0.750	0.867	0.907	0.836	0.886
	**Rad_Delta**	**0.910**	**0.8199–0.9993**	**0.907**	**0.833**	**0.929**	**0.789**	**0.929**	**0.907**	**0.885**	**0.918**
Training	DL_Pre	0.904	0.8575–0.9512	0.825	0.803	0.846	0.778	0.846	0.825	0.816	0.835
	DL_Post	0.971	0.9398-1.0000	0.925	0.951	0.961	0.906	0.961	0.925	0.936	0.943
	**DL_Delta**	**0.908**	**0.8517–0.9647**	**0.937**	**0.852**	**0.893**	**0.912**	**0.893**	**0.937**	**0.901**	**0.915**
Validation	DL_Pre	0.738	0.6057–0.8698	0.837	0.556	0.818	0.588	0.818	0.837	0.754	0.828
	DL_Post	0.804	0.6732–0.9340	0.791	0.722	0.872	0.591	0.872	0.791	0.770	0.829
	**DL_Delta**	**0.835**	**0.7021–0.9672**	**0.884**	**0.722**	**0.884**	**0.722**	**0.884**	**0.884**	**0.836**	**0.884**
Training	DL_Rad_Pre	0.934	0.8942–0.9745	0.887	0.836	0.877	0.850	0.877	0.887	0.865	0.882
	DL_Rad_Post	0.921	0.8679–0.9747	0.925	0.836	0.881	0.895	0.881	0.925	0.887	0.902
	**DL_Rad_Delta**	**0.941**	**0.8926–0.9893**	**0.937**	**0.869**	**0.904**	**0.914**	**0.904**	**0.937**	**0.908**	**0.920**
Validation	DL_Rad_Pre	0.695	0.5393–0.8509	0.628	0.778	0.871	0.467	0.871	0.628	0.672	0.730
	DL_Rad_Post	0.837	0.7385–0.9360	0.558	1.000	1.000	0.486	1.000	0.558	0.689	0.716
	**DL_Rad_Delta**	**0.925**	**0.8520–0.9982**	**0.837**	**0.944**	**0.973**	**0.708**	**0.973**	**0.837**	**0.869**	**0.900**

Delta, Pre + Post; PPV, positive pretictive value; NPV, negative pretictive value; CI, confidence interval; AUC, area under the curve

## Conclusion

We developed an integrated model integrating multimodal pre- and post- longitudinal CE-MRI images to predict long-term tumor response in HCC patients undergoing DEB-TACE. This model can assist clinicians in making better post-treatment decisions, such as transitioning to systemic therapy, selective internal radiation therapy, liver resection, or transplantation. Future prospective studies with external validation will further strengthen the clinical utility of this model and explore its applicability across diverse populations.

## Supplementary Information

Below is the link to the electronic supplementary material.

Supplementary Material 1

## Data Availability

No datasets were generated or analysed during the current study.

## Abbreviations

AFP: Alpha-fetoprotein
AP: Arterial phase
AUC: Area under the curve
CE-MRI: Contrast-enhanced magnetic resonance imaging
CI: Confidence interval
CR: Complete response
DEB-TACE: Drug-eluting beads transarterial chemoembolization
DL: Deep learning
DLF: Deep learning features
DP: Delayed phase
DRF: Delta radiomics features
EASL: European Association for the Study of the Liver
HCC: Hepatocellular carcinoma
ICC: Intraclass correlation coefficient
LASSO: Least absolute shrinkage and selection operator
ML: Machine learning
mRECIST: modified Response Evaluation Criteria in Solid Tumors
MRMR: Minimum redundancy maximum relevance
NGTDM: Neighboring gray-tone difference matrix
NPV: Negative predictive value
NR: Non-response
OR: Objective response
PD: Progressive disease
PPV: Positive predictive value
PR: Partial response
PVP: Portal venous phase
ROC: Receiver operating characteristic curve
ROI: Region of interest
SD: Stable disease
SDF: Siamese delta feature
SHAP: Shapley additive explanations
SVM: Support vector machine
T1WI: T1-weighted imaging
