# Supplementary material for: Longitudinal CE-MRI-based Siamese network with machine learning to predict tumor response in HCC after DEB-TACE
Source: Cancer Imaging. 2025 Aug 19;25:104. doi: 10.1186/s40644-025-00926-5 (PMC12366127; doi:10.1186/s40644-025-00926-5)
Supplement: Supplementary file 1 — Supplementary Material 1 [file 40644_2025_926_MOESM1_ESM.docx]

**Supplemental Digital Content**

**S1.**

**
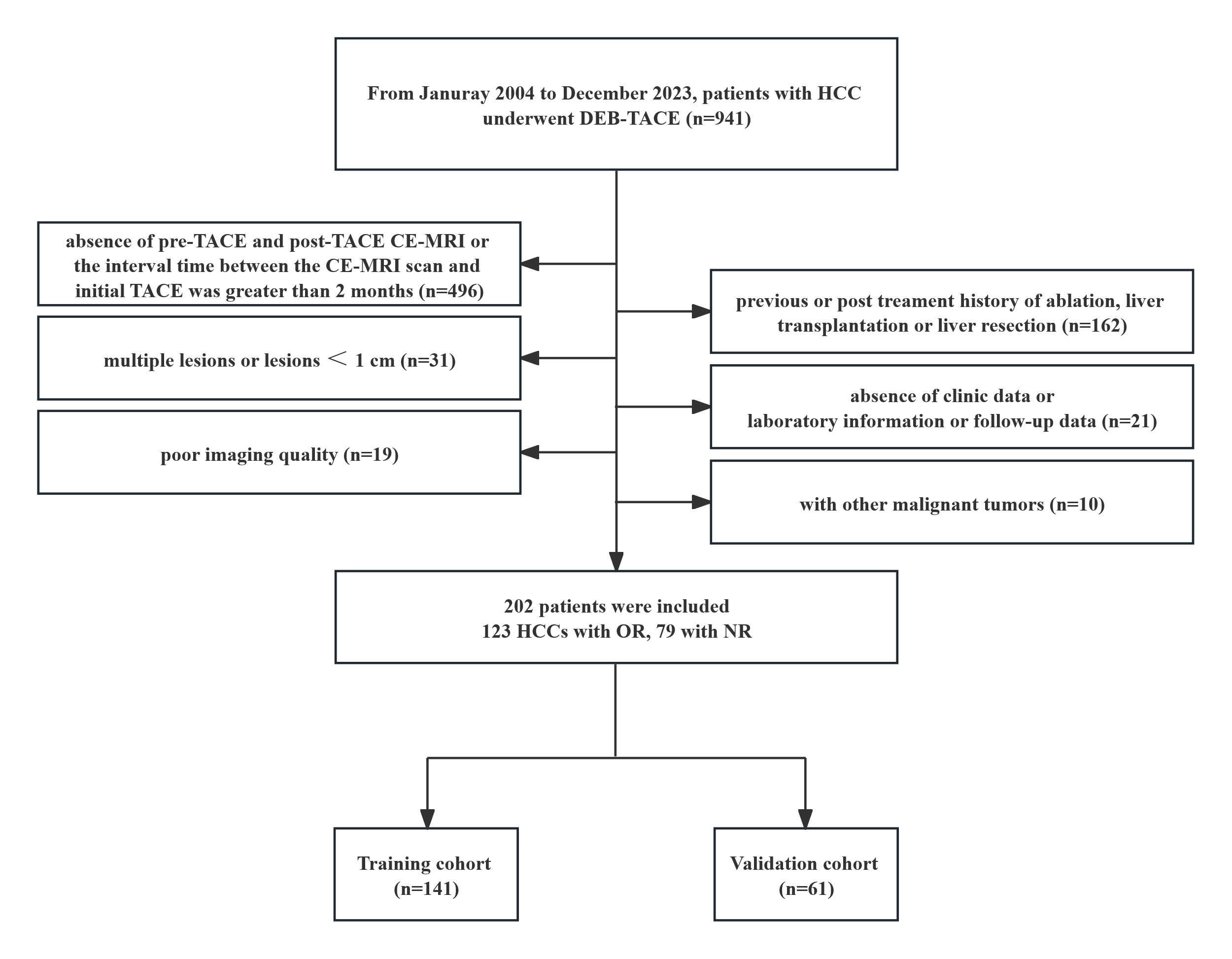
**

**Figure S1. Flowchart shows inclusion and exclusion criteria.**

The inclusion criteria were:

1. Histologically confirmed or radiologically diagnosed HCC, according to the European Association for the Study of the Liver (EASL) guidelines;
2. DEB-TACE as the initial treatment;
3. Having undergone three subsequent CE-MRI examinations: within two months prior and after the first DEB-TACE, as well as within 6 months after the second CE-MRI scan;
4. Complete clinical and laboratory data accessible for analysis.

The exclusion criteria were:

1. Prior or subsequent anti-tumor treatments aside from DEB-TACE (e.g. ablation, transplantation, or resection);
2. Systemic treatments (e.g. targeted therapy or immunotherapy) administered between the DEB-TACE and the third CE-MRI scan;
3. Incomplete or non-standard DEB-TACE procedures;
4. Poor-quality CE-MRI scans or absence of CE-MRI data;
5. Concurrent malignancies;
6. Diffuse or infiltrative disease, or multiple lesions with more than 6 nodules;
7. Maximum lesion size < 1 cm；
8. Absence of clinic data or laboratory information or follow-up data.

Among them, those without CE-MRI or with an interval greater than two months between CE-MRI and the first DEB-TACE were excluded (n=496). Patients presenting multiple lesions or lesion sizes smaller than 1 cm were also excluded (n=31), as were those with poor imaging quality (n=19). We further excluded patients who had undergone other treatments, such as ablation, liver transplantation, or liver resection, either before or after the DEB-TACE (n=162), as well as those lacking necessary clinical, laboratory, or follow-up data (n=21). Lastly, patients with other malignant tumors were removed (n=10).

After applying all these criteria, a total of 202 patients remained eligible for the study. Patients were randomly assigned to either the training or testing cohort in a 7:3 ratio, used for training and validating the DL and ML models. Data partitions were disjoint at the patient level, meaning that all data (including imaging and clinical features) from a single patient were assigned to only one partition (training and validation set) to prevent data leakage and ensure model generalizability.

**S2. Detailed description of DEB-TACE procedure**

All DEB-TACE procedures were performed by experienced interventional radiologists with a minimum of 10 years of clinical practice. The procedures were conducted under sterile conditions with patients positioned on a Digital Subtraction Angiography (DSA) table (Artis Zee, Siemens Healthineers, Erlangen, Germany) under local anesthesia. The procedure began with a retrograde puncture of the right common femoral artery (CFA) using a hollow needle, followed by the insertion of a short 5F sheath (Terumo, Japan) guided by a J-wire. A 5F Sidewinder catheter (COOK Medical, USA) was then advanced over the J-wire and positioned in the aortic arch. Subsequently, the catheter was maneuvered into the target celiac trunk (TC) during retraction, and coeliacography was performed to identify the likely feeder vessels. Next, a 2.4F Direxion Straight microcatheter (Boston Scientific, MA, USA) preloaded with a Fathom wire (Boston Scientific, MA, USA) was advanced into the right hepatic artery. From this position, Dyna-CT imaging with contrast medium was performed to identify the tumor-feeding vessels. Superselective probing of the feeder vessel was achieved, followed by the administration of a chemotherapeutic-contrast agent mixture in a superselective position until the second stasis was reached. Afterward, the catheter and sheath were carefully retracted, and manual compression was applied for 10 minutes, followed by the placement of a pressure bandage.

Typical Medication:

1. 15 mg piritramide intravenously i.v.
2. 1 mg granisetron i.v.
3. 10 ml Xylonest subcutaneously s.c.

Chemotherapeutic Agent/Embolizate:

A mixture of 2 ml DC Bead microspheres (Biocompatibles UK Ltd/Boston Scientific, MA, USA) or Embozene TANDEM microspheres (CeloNova Biosciences/Boston Scientific, MA, USA) loaded with 75 mg doxorubicin (70–100 µm), combined with 10 ml contrast medium and 5 ml NaCl.

Post-procedure Care:

1. Fasting for 2 hours.
2. Strict bed rest for 6 hours, followed by restricted bed rest for an additional 10 hours.
3. Maintenance of a pressure bandage for 16 hours.
4. Administration of analgesics and antiemetics as needed.

**S3. Table S1. Modified Response Evaluation Criteria in Solid Tumors (mRECIST1.1) Assessment for HCC**

| **Tumor Response** | **Description** |
| --- | --- |
| Complete response, CR | Disappearance of any intratumoral arterial enhancement in all target lesions |
| Partial response, PR | At least a 30% decrease in the sum of diameters of viable (enhancement in the arterial phase) target lesions, taking as reference the baseline sum of the diameters of target lesions |
| Stable disease, SD | Any cases that do not qualify for either partial response or progressive disease |
| Progressive disease, PD | An increase of at least 20% in the sum of the diameters of viable (enhancing) target lesions, taking as reference the smallest sum of the diameters of viable (enhancing) target lesions recorded since treatment started |

**S4. Magnetic resonance imaging sequences and parameters**

The contrast-enhanced imaging included arterial, portal venous, and delayed phases, acquired at 25 seconds, 60 seconds, and 180 seconds after administering Gd-diethylenetriamine pentaacetic acid (Gd-DTPA) (Bayer Schering Pharma AG, Germany). The contrast agent was delivered through the median cubital vein at a rate of 2.5 ml/s, with the dosage adjusted to 0.1 mmol/kg based on the patient’s weight.

Poor-quality images were defined as those exhibiting any of the following: (1) significant motion artifacts; (2) inadequate contrast enhancement of the liver or lesion; or (3) incomplete coverage of the liver. Image quality was independently assessed by the same two radiologists, and only those images meeting acceptable standards were included in the analysis. In cases of disagreement regarding segmentation or image quality, a senior radiologist with more than 10 years of clinical experience reviewed the case and made the final determination.

**Table S2. MRI scanning parameters for the patients**

| Field strength | Echo time (ms) | Repetition time (ms) | FOV (cm^2^) | Matrix size | NEX | Section thickness (mm) | Flip Angle |
| --- | --- | --- | --- | --- | --- | --- | --- |
| 1.5T | 2.1 | 4.7 | 33×45 | 288×384 | 1.0 | 3.5 | 10 |
| 3.0T | 1.3 | 3.7 | 36×44 | 312×384 | 1.0 | 3.0 | 9 |

FOV, field of view; NEX, number of excitation.

**S5. Image processing**

All imaging data were de-identified prior to analysis, with all patient identifiers removed from the DICOM headers and image data, in accordance with institutional and regulatory guidelines. To preprocess MRI sequences, a series of crucial steps were undertaken, including resampling, intensity normalization, and N4 bias field correction. Initially, resampling was applied to standardize the voxel dimensions across all MRI images, ensuring spatial consistency and comparability between subjects. This resampling was conducted using the SimpleITK library in Python, with a target spacing of 1.0 × 1.0 × 1.0 mm^3^. Interpolation methods were tailored to the specific MRI sequence type, thereby minimizing the introduction of artifacts and maintaining anatomical fidelity.

Given the broad range of intensity values inherent in MRI images, z-score normalization was performed to harmonize the intensity distributions. This procedure transformed the intensity values into a standard normal distribution characterized by a mean (μ) of 0 and a standard deviation (σ) of 1. The normalization process was crucial for mitigating intensity variations among different scans and enabling more robust analysis across datasets. The normalized intensity (z-score) for each voxel (x) was computed using the following formula:

$$z= \frac{x-\mu}{\sigma}$$

Finally, N4 bias field correction was applied to rectify intensity inhomogeneities caused by variations in magnetic field strength during image acquisition. This step utilized the N4ITK algorithm, which has been shown to effectively correct low-frequency intensity non-uniformity, thereby enhancing the quality of subsequent segmentation and quantitative analysis. The implementation was executed via the SimpleITK package (version 4.0.1), based on the method described by Tustison et al. (“N4ITK: Nick's N3 ITK Implementation For MRI Bias Field Correction”, http://hdl.handle.net/10380/3053). This bias correction step was integral for ensuring the reliability of downstream radiomics and machine learning analyses by improving the consistency of tissue intensity profiles within and across subjects.

**S6. Details of radiomics features**

Feature extraction was conducted using Pyradiomics ([https://pyradiomics.readthedocs.io/en/latest/](https://pyradiomics.readthedocs.io/en/latest/)). The extracted radiomic features were categorized into seven distinct types: (i) 14 shape-based features; (ii) 234 first-order features; (iii) 182 features from the gray level dependence matrix (GLDM); (iv) 208 features from the gray level size zone matrix (GLSZM); (v) 65 features from the neighboring gray-tone difference matrix (NGTDM); (vi) 208 features from the gray level run-length matrix (GLRLM); and (vii) 286 features from the gray level co-occurrence matrix (GLCM). This resulted in a total of 1,197 features extracted from each ROI and its corresponding CE-MRI sequence.

1. **Shape based features**

In this group of features, we included descriptors of the three-dimensional shape and size of the tumor region. Let in the following definitions V denote the volume and A the surface area of the volume of interest. *N_v_* represent the number of voxels included in the ROI. *N_f_* represent the number of faces (triangles) defining the Mesh. We determined the following shape and size based features:

1. **Elongation:**

$$elongation = \sqrt{\frac{\lambda_{minor}}{\lambda_{major}}}$$

Here, *λ_major_* and *λ_minor_* are the lengths of the largest and second largest principal component axes.

1. **Flatness:**

$$flatness = \sqrt{\frac{\lambda_{least}}{\lambda_{major}}}$$

Here, *λ_major_* and *λ_least_* are the lengths of the largest and smallest principal component axes. The values range between 1 (non-flat, sphere-like) and 0 (a flat object, or single-slice segmentation).

1. **Least Axis Length:**

$least axis=4\sqrt{\lambda_{least}}$

This feature yield the smallest axis length of the ROI-enclosing ellipsoid and is calculated using the largest principal component *λ_least_*.

1. **Major Axis Length:**

$$major axis=4\sqrt{\lambda_{major}}$$

This feature yield the largest axis length of the ROI-enclosing ellipsoid and is calculated using the largest principal component *λ_major_*.

1. **Maximum 2D diameter (Column):**

Maximum 2D diameter (Column) is defined as the largest pairwise Euclidean distance between tumor surface mesh vertices in the row-slice (usually the coronal) plane.

1. **Maximum 2D diameter (Row):**

Maximum 2D diameter (Row) is defined as the largest pairwise Euclidean distance between tumor surface mesh vertices in the column-slice (usually the sagittal) plane.

1. **Maximum 2D diameter (Slice):**

Maximum 2D diameter (Slice) is defined as the largest pairwise Euclidean distance between tumor surface mesh vertices in the row-column (generally the axial) plane.

1. **Maximum 3D diameter:**

Maximum 3D diameter is defined as the largest pairwise Euclidean distance between tumor surface mesh vertices.

Also known as Feret Diameter.

1. **Mesh Volume:**

$$V=\sum_{i=1}^{N_{f}} \frac{Oa_{i}\cdot(Ob_{i}\times Oc_{i})}{6}$$

The volume of the ROI *V* is calculated from the triangle mesh of the ROI. For each face *i* in the mesh, defined by points *a_i_*, *b_i_* and *c_i_*, the (signed) volume *V* of the tetrahedron defined by that face and the origin of the image (*O*) is calculated.

1. **Minor Axis Length:**

$$minor axis =4\sqrt{\lambda_{minor}}$$

This feature yield the second-largest axis length of the ROI-enclosing ellipsoid and is calculated using the largest principal component *λ_minor_*.

1. **Sphericity:**

$$sphericity=\frac{\pi^{\frac{1}{3}}{(6V)}^{\frac{2}{3}}}{A}$$

2. **Surface area:**

The surface area is calculated by triangulation (i.e. dividing the surface into connected triangles) and is defined as:

$$A=\sum_{i=1}^{N} \frac{1}{2}\left| a_{i}b_{i}\times a_{ic_{i}} \right|$$

*a_i_b_i_* and *a_i_c_i_* are edges of the *i*^th^ triangle in the mesh, formed by vertices *a_i_*, *b_i_* and *c_i_*.

1. **Surface to volume ratio:**

$$surface to volume ratio=\frac{A}{V}$$

2. **Voxel Volume:** The volume (*V*) of the tumor is determined by counting the number of pixels in the tumor region and multiplying this value by the voxel size.
3. **First order statistical features**

The following 18 statistical features were extracted.

Let:

$X$be a set of Np voxels included in the ROI. P($i$) be the first order histogram with Ng discrete intensity levels, where *N_g_* is the number of non-zero bins, equally spaced from 0 with a width defined in the binwidth parameter. P($i$) be the normalized first order histogram and equal to $\frac{\mathbf{P}(i)}{\boldsymbol{N}_{p}}$.

1. **10Percentile:**

The 10^th^ percentile of $X$.

1. **90Percentile:**

The 90^th^ percentile of $X$.

1. **Energy:**

$$energy=\sum_{i=1}^{N_{p}} {(\boldsymbol{X}(i)+c)}^{2}$$

Here, *c* is optional value, defined by voxelArrayShift, which shifts the intensities to prevent negative values in $X$.

1. **Entropy:**

$$entropy=-\sum_{i=1}^{N_{g}} P\left( i \right){log}_{2} P\left( i \right)+\epsilon$$

Here, ϵ is an arbitrarily small positive number (≈2.2×10^−16^).

1. **Inter-quartile Range:**

$$interquartile range= \boldsymbol{P}_{75} -\boldsymbol{P}_{25}$$

Here **P**_25_ and **P**_75_ are the 25^th^ and 75^th^ percentile of the image array, respectively.

1. **Kurtosis:**

$$kurtosis=\frac{\frac{1}{N_{p}}\sum_{i=1}^{N_{p}} \left( X\left( i \right)-\bar{X} \right)^{4}}{\left( \sqrt{\frac{1}{N_{p}}\sum_{i=1}^{N} \left( X\left( i \right)-\bar{X} \right)^{2}} \right)^{2}}$$

Where $\overline{X}$ is the mean of $X$.

1. **Maximum:**

The maximum intensity value of $X$.

1. **Mean:**

$$mean=\frac{1}{N}\sum_{i}^{N} X(i)$$

2. **Mean Absolute Deviation:**

The mean of the absolute deviations of all voxel intensities around the mean intensity value.

1. **Median:**

The median intensity value of$X$.

1. **Minimum:**

The minimum intensity value of $X$.

1. **Range:**

The range of intensity values of$X$.

1. **Robust Mean Absolute Deviation:**

$$rMAD=\frac{1}{N_{10-90}}\sum_{i=1}^{N_{10-90}} \left| X_{10-90}(i) - \overline{X}_{10-90} \right|$$

2. **Root Mean Squared:**

$$RMS=\sqrt{\frac{\sum_{i}^{N} {X(i)}^{2}}{N}}$$

3. **Skewness:**

$$skewness=\frac{\frac{1}{N}\sum_{i=1}^{N} \left( X\left( i \right)-\bar{X} \right)^{3}}{\left( \sqrt{\frac{1}{N}\sum_{i=1}^{N} \left( X\left( i \right)-\bar{X} \right)^{2}} \right)^{3}}$$

Where $\overline{X}$ is the mean of $X$.

1. **Total Energy:**

$$total energy=V_{voxel}\sum_{i=1}^{N_{p}} {(\boldsymbol{X}(i)+c)}^{2}$$

Here, *c* is optional value, defined by voxelArrayShift, which shifts the intensities to prevent negative values in $X$.

1. **Uniformity:**

$$uniformity=\sum_{i=1}^{N_{g}} {P\left( i \right)}^{2}$$

2. **Variance:**

$$variance=\frac{1}{N_{p}}\sum_{i=1}^{N_{p}} {(X\left( i \right)-\bar{X})}^{2}$$

Where $\overline{X}$ is the mean of $X$.

1. **Texture features**

Second order statistic texture features, and higher order statistic texture features were extracted. 110 second order statistic texture features could be calculated from the Gray Level Co-occurrence Matrix (GLCM). 255 high order statistic texture features were calculated from the Gray Level Size Zone Matrix (GLSZM), Gray Level Run Length Matrix (GLRLM), and Neighborhood Gray Tone Difference Matrix (NGTDM). All of the GLCM, GLSZM, GLRLM, and NGTDM based texture feature were calculated using a 2D analysis and then averaged for all slices within the three-dimensional tumor volume.

*Gray-Level Co-Occurrence Matrix based features (GLCM)*

GLCM based features were second-order statistical texture features, which are defined as a matrix M (*i, j; δ, θ*) to indicate the relative frequency with intensity values of pixels (*i* and *j*) at the distance of δ in direction θ.

Let:

ϵ be an arbitrarily small positive number(≈2.2×10^−16^).

P($i$,$j$) be the co-occurrence matrix for an arbitrary *δ* and *θ.*

*p_x_*($i$,$j$)be the normalized co-occurrence matrix and equal to $\frac{\mathbf{P}(i,j)}{\sum\mathbf{P}(i,j)}$.

*N*_g_ be the number of discrete intensity levels in the image.

$p_{x}(i)=\sum_{j=1}^{N_{g}} p(i,j)$be the marginal row probabilities.

$p_{y}(j)=\sum_{j=1}^{N_{g}} p(i,j)$be the marginal column probabilities.

$\mu_{x}$ be the mean gray level intensity of *p_x_* and defined as $\mu_{x}=\sum_{i=1}^{N_{g}} p_{x}(i)i$.

$\mu_{y}$ be the mean gray level intensity of *p_y_* and defined as $\mu_{y}=\sum_{j=1}^{N_{g}} p_{x}(j)j$.

$\sigma_{x}$ be the standard deviation of *p_x_*.

$\sigma_{y}$ be the standard deviation of *p_y_*.

$P_{x+y}(k)=\sum_{i=1}^{N_{g}} \sum_{j=1}^{N_{g}} p(i,j)$, where $i+j=k$, and $k=2,3,...,2N_{g}$.

$P_{x-y}(k)=\sum_{i=1}^{N_{g}} \sum_{j=1}^{N_{g}} p(i,j)$, where $\left| i-j \right|=k$, and $k=0,1,...,N_{g}-1$.

$HX=-\sum_{i=1}^{N_{g}} p_{x}(i){log}_{2}(p_{x}(i)+\epsilon$ be the entropy of $p_{x}$.

$HY=-\sum_{i=1}^{N_{g}} p_{y}(i){log}_{2}(p_{y}(i)+\epsilon$ be the entropy of $p_{y}$.

$HXY=-\sum_{i=1}^{N_{g}} \sum_{j=1}^{N_{g}} p(i,j){log}_{2}(p(i,j)+\epsilon)$be the entropy of *p($i,j$).*

$HXY1=-\sum_{i=1}^{N_{g}} \sum_{j=1}^{N_{g}} p(i,j){log}_{2}(p_{x}(i)p_{y}(j)+\epsilon)$*.*

$HXY2=-\sum_{i=1}^{N_{g}} \sum_{j=1}^{N_{g}} p_{x}(i)p_{y}(j){log}_{2}(p_{x}(i)p_{y}(j)+\epsilon)$*.*

1. **Auto-correlation:**

$$\sum_{i=1}^{N_{g}} \sum_{j=1}^{N_{g}} p(i,j)ij$$

2. **Cluster Prominence:**

$$\sum_{i=1}^{N_{g}} \sum_{j=1}^{N_{g}} {(i+j-\mu_{x}-\mu_{y})}^{4}p(i,j)$$

3. **Cluster Shade:**

$$\sum_{i=1}^{N_{g}} \sum_{j=1}^{N_{g}} {(i+j-\mu_{x}-\mu_{y})}^{3}p(i,j)$$

4. **Cluster Tendency:**

$$\sum_{i=1}^{N_{g}} \sum_{j=1}^{N_{g}} {(i+j-\mu_{x}-\mu_{y})}^{2}p(i,j)$$

5. **Contrast:**

$$\sum_{i=1}^{N_{g}} \sum_{j=1}^{N_{g}} {(i-j)}^{2}p(i,j)$$

6. **Correlation:**

$$\frac{\sum_{i=1}^{N_{g}} \sum_{j=1}^{N_{g}} p(i,j)ij-\mu_{x}\mu_{y}}{\sigma_{x}(i)\sigma_{y}(j)}$$

7. **Difference Average:**

$$\sum_{k=0}^{N_{g}-1} {kp}_{x-y}(k)$$

8. **Difference Entropy:**

$$\sum_{k=0}^{N_{g}-1} p_{x-y}(k){log}_{2}(p_{x-y}(k)+\epsilon)$$

9. **Difference Variance:**

$$\sum_{k=0}^{N_{g}-1} {{(k-DA)}^{2}p}_{x-y}(k)$$

10. **Inverse Difference(ID):**

$$\sum_{k=0}^{N_{g}-1} \frac{p_{x-y}(k)}{1+k}$$

11. **Inverse Difference Moment(IDM):**

$$\sum_{k=0}^{N_{g}-1} \frac{p_{x-y}(k)}{1+k^{2}}$$

12. **Inverse Difference Moment Normalized(IDMN):**

$$\sum_{k=0}^{N_{g}-1} \frac{p_{x-y}(k)}{1+(\frac{k^{2}}{N_{g}^{2}})}$$

13. **Inverse Difference Normalized(IDN):**

$$\sum_{k=0}^{N_{g}-1} \frac{p_{x-y}(k)}{1+(\frac{k}{N_{g}})}$$

14. **Informational Measure of Correlation 1(IMC1):**

$$\frac{HXY-HXY1}{max\{HX,HY\}}$$

15. **Informational Measure of Correlation 2(IMC2):**

$$\sqrt{1-e^{-2(HXY2-HXY)}}$$

16. **Inverse Variance:**

$$\sum_{k=1}^{N_{g}-1} \frac{p_{x-y}(k)}{k^{2}}$$

17. **Joint Average:**

$$\mu_{x}=\sum_{i=1}^{N_{g}} \sum_{j=1}^{N_{g}} p(i,j)i$$

18. **Joint Energy:**

$$\sum_{i=1}^{N_{g}} \sum_{j=1}^{N_{g}} {(p(i,j))}^{2}$$

19. **Joint Entropy:**

$$-\sum_{i=1}^{N_{g}} \sum_{j=1}^{N_{g}} p(i,j){log}_{2}(p(i,j)+\epsilon)$$

20. **Maximum Probability:**

$$maximum probability=max(p(i,j))$$

21. **Sum Entropy:**

$$\sum_{k=2}^{{2N}_{g}} p_{x+y}\left( k \right)k$$

22. **Sum Squares:**

$$\sum_{i=1}^{N_{g}} \sum_{j=1}^{N_{g}} {(i-\mu_{x})}^{2}p(i,j)$$

*Gray Level Run Length Matrix based features (GLRLM)*

GLRLM based features were high-order statistical texture feature, which were defined as *P*(*i, j; θ*) to indicate the number of times j and gray level i appear consecutively in the direction *θ*.

Let:

*N_g_* be the number of discrete intensity values in the ROI.

*Nr* be the number of discrete run lengths in the ROI.

*Np* be the number of voxels in the ROI.

*Nr*(*θ*) be the number of runs in the image along angle θ, which is equal to $-\sum_{i=1}^{N_{g}} \sum_{j=1}^{N_{r}} \mathbf{P}(i,j\left| \theta\right.)$ and $1\leq N_{r}(\theta)\leq N_{p}$

 $\mathbf{P}(i,j\left| \theta\right.)$ be the run length matrix for an arbitrary direction $\theta$.

 $p(i,j\left| \theta\right.)$ be the normalized run length matrix, defined as $p(i,j\left| \theta\right.)=\frac{\mathbf{P}(i,j\left| \theta\right.)}{N_{r}(\theta)}$

1. **Gray-Level Non-Uniformity(GLN):**

$$\frac{\sum_{i=1}^{N_{g}} \left( \sum_{j=1}^{N_{r}} \mathbf{P}(i,j\left| \theta\right.) \right)^{2}}{N_{r}(\theta)}$$

2. **Gray-Level Non-Uniformity Normalize(GLNN):**

$$\frac{\sum_{i=1}^{N_{g}} \left( \sum_{j=1}^{N_{r}} \mathbf{P}(i,j\left| \theta\right.) \right)^{2}}{{N_{r}(\theta)}^{2}}$$

3. **Gray-Level Variance(GLV):**

$$\sum_{i=1}^{N_{g}} \sum_{j=1}^{N_{r}} {p(i,j\left| \theta\right.)(i-\mu)}^{2}$$

Here, $\mu=\sum_{i=1}^{N_{g}} \sum_{j=1}^{N_{r}} p(i,j\left| \theta\right.)i$

1. **High Gray-Level Run Emphasis(HGLRE):**

$$\frac{\sum_{i=1}^{N_{g}} \sum_{j=1}^{N_{r}} \mathbf{P}(i,j\left| \theta\right.)i^{2}}{N_{r}(\theta)}$$

2. **Long Run Emphasis(LRE):**

$$\frac{\sum_{i=1}^{N_{g}} \sum_{j=1}^{N_{r}} \mathbf{P}(i,j\left| \theta\right.)j^{2}}{N_{r}(\theta)}$$

3. **Long Run High Gray-Level Emphasis(LRHGLE):**

$$\frac{\sum_{i=1}^{N_{g}} \sum_{j=1}^{N_{r}} \mathbf{P}(i,j\left| \theta\right.)i^{2}j^{2}}{N_{r}(\theta)}$$

4. **Long Run Low Gray-Level Emphasis(LRLGLE):**

$$\frac{\sum_{i=1}^{N_{g}} \sum_{j=1}^{N_{r}} \frac{\mathbf{P}(i,j\left| \theta\right.)j^{2}}{i^{2}}}{N_{r}(\theta)}$$

5. **Low Gray-Level Run Emphasis(LGLRE):**

$$\frac{\sum_{i=1}^{N_{g}} \sum_{j=1}^{N_{r}} \frac{\mathbf{P}(i,j\left| \theta\right.)}{i^{2}}}{N_{r}(\theta)}$$

6. **Run Entropy(RE):**

$$-\sum_{i=1}^{N_{g}} \sum_{j=1}^{N_{r}} p(i,j\left| \theta\right.){log}_{2}(p(i,j\left| \theta\right.)+\epsilon)$$

7. **Run Length Non-Uniformity(RLN):**

$$\frac{\sum_{j=1}^{N_{r}} \left( \sum_{i=1}^{N_{g}} \mathbf{P}(i,j\left| \theta\right.) \right)^{2}}{N_{r}(\theta)}$$

8. **Run Length Non-Uniformity Normalized(RLNN):**

$$\frac{\sum_{j=1}^{N_{r}} \left( \sum_{i=1}^{N_{g}} \mathbf{P}(i,j\left| \theta\right.) \right)^{2}}{{N_{r}(\theta)}^{2}}$$

9. **Run Percentage(RP):**

$$\frac{N_{r}(\theta)}{N_{p}}$$

10. **Run Variance(RV):**

$$\sum_{i=1}^{N_{g}} \sum_{j=1}^{N_{r}} {p(i,j\left| \theta\right.)(j-\mu)}^{2}$$

Here, $\mu=\sum_{i=1}^{N_{g}} \sum_{j=1}^{N_{r}} p(i,j\left| \theta\right.)i$

1. **Short Run Emphasis(SRE):**

$$\frac{\sum_{i=1}^{N_{g}} \sum_{j=1}^{N_{r}} \frac{\mathbf{P}(i,j\left| \theta\right.)}{j^{2}}}{N_{r}(\theta)}$$

2. **Short Run High Gray-Level Emphasis(SRHGLE):**

$$\frac{\sum_{i=1}^{N_{g}} \sum_{j=1}^{N_{r}} \frac{\mathbf{P}(i,j\left| \theta\right.)i^{2}}{j^{2}}}{N_{r}(\theta)}$$

3. **Short Run Low Gray-Level Emphasis(SRLGLE):**

$$\frac{\sum_{i=1}^{N_{g}} \sum_{j=1}^{N_{r}} \frac{\mathbf{P}(i,j\left| \theta\right.)}{i^{2}j^{2}}}{N_{r}(\theta)}$$

*Gray Level Size Zone Matrix based features (GLSZM)*

GLSZM based features were high-order statistical texture features, which were defined as *P*(*i, j*) to indicate the areas of size *j* and gray level *i*.

Let:

*P*(*i, j*) be the size zone of matrix *P.*

*N_g_* be the number of discrete intensity values.

*N_s_* be the number of different areas sizes.

*N_p_* be the number of voxels in the ROI.

*N_z_* be the number of zones in the ROI, which is equal to $\sum_{i=1}^{N_{g}} \sum_{j=1}^{N_{s}} \mathbf{P}(i,j)$ and ${1\leq N}_{z}\leq N_{p}$

1. **Gray-Level Non-Uniformity(GLN):**

$$\frac{\sum_{i=1}^{N_{g}} \left( \sum_{j=1}^{N_{s}} \mathbf{P}(i,j) \right)^{2}}{N_{z}}$$

2. **Gray-Level Non-Uniformity Normalized(GLNN):**

$$\frac{\sum_{i=1}^{N_{g}} \left( \sum_{j=1}^{N_{s}} \mathbf{P}(i,j) \right)^{2}}{N_{z}^{2}}$$

3. **Gray-Level Variance(GLV):**

$$\sum_{i=1}^{N_{g}} \sum_{j=1}^{N_{s}} {p(i,j)(i-\mu)}^{2}$$

Here, $\mu=\sum_{i=1}^{N_{g}} \sum_{j=1}^{N_{s}} p(i,j)i$

1. **High Gray-Level Zone Emphasis(HGLZE):**

$$\frac{\sum_{i=1}^{N_{g}} \sum_{j=1}^{N_{s}} \mathbf{P}(i,j)i^{2}}{N_{z}}$$

2. **Large Area Emphasis(LAE):**

$$\frac{\sum_{i=1}^{N_{g}} \sum_{j=1}^{N_{s}} \mathbf{P}(i,j)j^{2}}{N_{z}}$$

3. **Large Area High Gray-Level Emphasis(LAHGLE):**

$$\frac{\sum_{i=1}^{N_{g}} \sum_{j=1}^{N_{s}} \mathbf{P}(i,j)i^{2}j^{2}}{N_{z}}$$

4. **Large Area Low Gray-Level Emphasis(LALGLE):**

$$\frac{\sum_{i=1}^{N_{g}} \sum_{j=1}^{N_{s}} \frac{\mathbf{P}(i,j)j^{2}}{i^{2}}}{N_{z}}$$

5. **Low Gray-Level Zone Emphasis(LGLZE):**

$$\frac{\sum_{i=1}^{N_{g}} \sum_{j=1}^{N_{s}} \frac{\mathbf{P}(i,j)}{i^{2}}}{N_{z}}$$

6. **Size Zone Non-Uniformity(SZN):**

$$\frac{\sum_{j=1}^{N_{s}} \left( \sum_{i=1}^{N_{j}} \mathbf{P}(i,j) \right)^{2}}{N_{z}}$$

7. **Size Zone Non-Uniformity Normalized(SZNN):**

$$\frac{\sum_{j=1}^{N_{s}} \left( \sum_{i=1}^{N_{j}} \mathbf{P}(i,j) \right)^{2}}{N_{z}^{2}}$$

8. **Small Area Emphasis(SAE):**

$$\frac{\sum_{i=1}^{N_{g}} \sum_{j=1}^{N_{s}} \frac{\mathbf{P}(i,j)}{j^{2}}}{N_{z}}$$

9. **Small Area High Gray-Level Emphasis(SAHGLE):**

$$\frac{\sum_{i=1}^{N_{g}} \sum_{j=1}^{N_{s}} \frac{\mathbf{P}(i,j)i^{2}}{j^{2}}}{N_{z}}$$

10. **Small Area Low Gray-Level Emphasis(SALGLE):**

$$\frac{\sum_{i=1}^{N_{g}} \sum_{j=1}^{N_{s}} \frac{\mathbf{P}(i,j)}{i^{2}j^{2}}}{N_{z}}$$

11. **Zone Entropy(ZE):**

$$-\sum_{i=1}^{N_{g}} \sum_{j=1}^{N_{s}} p(i,j){log}_{2}(p(i,j)+\epsilon)$$

12. **Zone Percentage(ZP):**

$$\frac{N_{z}}{N_{p}}$$

13. **Zone Variance(ZV):**

$$\sum_{i=1}^{N_{g}} \sum_{j=1}^{N_{s}} {p(i,j)(j-\mu)}^{2}$$

Here, $\mu=\sum_{i=1}^{N_{g}} \sum_{j=1}^{N_{s}} p(i,j)i$

*Neighborhood Gray Tone Difference Matrix based features (NGTDM)*

NGTDM based features were high-order statistical texture features, which were defined as *S(i)* to indicate the sum of the absolute value between gray intensity level i and it’s neighbors’ average intensity.

Let:

*S(i)* be the sum of absolute value between gray intensity level i and its neighbors’ average intensity,

*C(i)* be the number of voxels with the gray intensity level I,

*N_g_* be the number of discrete intensity values.

1. **Busyness:**

$$\frac{\sum_{i=1}^{N_{g}} p_{i}s_{i}}{\sum_{i=1}^{N_{g}} \sum_{j=1}^{N_{g}} \left| {ip}_{i}-jp_{j} \right|}$$

where $p_{i}\neq0,p_{j}\neq0$

1. **Coarseness:**

$$\frac{1}{\sum_{i=1}^{N_{g}} p_{i}s_{i}}$$

2. **Complexity:**

$$\frac{1}{N_{v,p}}\sum_{i=1}^{N_{g}} \sum_{j=1}^{N_{g}} \left| i-j \right|\frac{p_{i}{s_{i}+p_{j}s}_{j}}{p_{i}+p_{j}}$$

where $p_{i}\neq0,p_{j}\neq0$

1. **Contrast:**

$$\left( \frac{1}{N_{g,p}\left( N_{g,p}-1 \right)}\sum_{i=1}^{N_{g}} \sum_{j=1}^{N_{g}} p_{i}p_{j}\left( i-j \right)^{2} \right)\left( \frac{1}{N_{v,p}}\sum_{i=1}^{N_{g}} s_{i} \right)$$

where $p_{i}\neq0,p_{j}\neq0$

1. **Strength:**

$$\frac{\sum_{i=1}^{N_{g}} \sum_{j=1}^{N_{g}} \left( p_{i}+p_{j} \right)\left( i-j \right)^{2}}{\sum_{i=1}^{N_{g}} s_{i}}$$

where $p_{i}\neq0,p_{j}\neq0$

### *Gray Level Dependence Matrix based features (GLDM)*

Gray Level Dependence Matrix (GLDM) based features are high-order statistical texture features, where ***P*(***i,j*) represents the number of times a voxel with gray level *i* has exactly *j* dependent neighboring voxels within a specified distance in the image.

Let:

*N_g_* be the number of discrete intensity values in the image.

*N_d_* be the number of discrete dependency sizes in the image.

*N_z_* be the number of dependency zones in the image, which is equal to$\sum_{i=1}^{N_{g}} \sum_{j=1}^{N_{d}} \mathbf{P}(i,j)$.

***P*(***i,j*) be the dependence matrix.

*p*(*i,j*) be the normalized dependence matrix, defined as $p(i,j)=\frac{\boldsymbol{P}\boldsymbol{(}i,j)}{N_{z}}$.

1. **Dependence Entropy(DE):**

$$-\sum_{i=1}^{N_{g}} \sum_{j=1}^{N_{d}} p(i,j){log}_{2}(p(i,j)+\epsilon)$$

2. **Dependence Non-Uniformity(DN):**

$$\frac{\sum_{i=1}^{N_{d}} \left( \sum_{i=1}^{N_{g}} \mathbf{P}(i,j) \right)^{2}}{N_{z}}$$

3. **Dependence Non-Uniformity Normalized(DNN):**

$$\frac{\sum_{j=1}^{N_{d}} \left( \sum_{i=1}^{N_{g}} \mathbf{P}(i,j) \right)^{2}}{N_{z}^{2}}$$

4. **Dependence Variance(DV):**

$$\sum_{i=1}^{N_{d}} \sum_{j=1}^{N_{d}} {p(i,j)(j-\mu)}^{2}$$

Here, $\mu=\sum_{i=1}^{N_{g}} \sum_{j=1}^{N_{d}} jp(i,j)$

1. **Gray-Level Non-Uniformity(GLN):**

$$\frac{\sum_{i=1}^{N_{g}} \left( \sum_{j=1}^{N_{d}} \mathbf{P}(i,j) \right)^{2}}{N_{z}}$$

2. **Gray-Level Variance(GLV):**

$$\sum_{i=1}^{N_{g}} \sum_{j=1}^{N_{d}} {p(i,j)(i-\mu)}^{2}$$

Here, $\mu=\sum_{i=1}^{N_{g}} \sum_{j=1}^{N_{d}} jp(i,j)$

1. **High Gray-Level Emphasis(HGLE):**

$$\frac{\sum_{i=1}^{N_{g}} \sum_{j=1}^{N_{d}} \mathbf{P}(i,j)i^{2}}{N_{z}}$$

2. **Large Dependence Emphasis(LDE):**

$$\frac{\sum_{i=1}^{N_{g}} \sum_{j=1}^{N_{d}} \mathbf{P}(i,j)j^{2}}{N_{z}}$$

3. **Large Dependence High Gray-Level Emphasis(LDHGLE):**

$$\frac{\sum_{i=1}^{N_{g}} \sum_{j=1}^{N_{d}} \mathbf{P}(i,j)i^{2}j^{2}}{N_{z}}$$

4. **Large Dependence Low Gray-Level Emphasis(LDLGLE):**

$$\frac{\sum_{i=1}^{N_{g}} \sum_{j=1}^{N_{d}} \frac{\mathbf{P}(i,j)j^{2}}{i^{2}}}{N_{z}}$$

5. **Low Gray-Level Emphasis(LGLE):**

$$\frac{\sum_{i=1}^{N_{g}} \sum_{j=1}^{N_{d}} \frac{\mathbf{P}(i,j)}{i^{2}}}{N_{z}}$$

6. **Small Dependence Emphasis(SDE):**

$$\frac{\sum_{i=1}^{N_{g}} \sum_{j=1}^{N_{d}} \frac{\mathbf{P}(i,j)}{i^{2}}}{N_{z}}$$

7. **Small Dependence High Gray-Level Emphasis(SDHGLE):**

$$\frac{\sum_{i=1}^{N_{g}} \sum_{j=1}^{N_{d}} \frac{\mathbf{P}(i,j)i^{2}}{j^{2}}}{N_{z}}$$

8. **Small Dependence Low Gray-Level Emphasis(SDLGLE):**

$$\frac{\sum_{i=1}^{N_{g}} \sum_{j=1}^{N_{d}} \frac{\mathbf{P}(i,j)}{i^{2}j^{2}}}{N_{z}}$$

**(4) Wavelet features: first order statistical and texture features of a wavelet filtered image.**

A total of 728 derived wavelet features were extracted for each sequence, with the Gaussian filter and a wavelet-based filter. These features were computed on the filtered images. The original image was filtered by 8 filters. For each image, the first order statistical and texture features were computed.

**S7. Deep learning network architecture and training**

1. **Architecture**
2. Siamese Network

The proposed Siamese network consists of two 2D ResNet50 models, each receiving preoperative and postoperative three-channel MRI images as input, specifically T1-weighted images in the arterial phase, portal venous phase, and delayed phase. Each ResNet50 model processes the input through an initial convolutional layer, followed by a max pooling layer. The network then passes through four residual blocks containing 3, 4, 6, and 3 bottleneck layers, respectively. Each bottleneck block includes a 1x1x1 convolution for dimension reduction, a 3x3x3 convolution for feature extraction, and a final 1x1 convolution to restore feature dimensions. Skip connections in each block add input features to the output, enhancing gradient flow and mitigating vanishing gradients. After the last residual block, global average pooling reduces the spatial dimensions while retaining feature depth, resulting in a 2048-dimensional feature vector. These feature vectors are independently generated for the preoperative and postoperative images and are used to predict tumor response.

1. Transformer Network

The fusion of these preoperative and postoperative features is performed using a Transformer network. The Transformer operates on the extracted feature vectors from both time points. The extracted features are first concatenated and projected into the Transformer’s input space. The Transformer’s architecture, with multi-head self-attention mechanisms and feed-forward layers, is then employed to model the interactions between preoperative and postoperative features. The self-attention mechanism enables the model to focus on different aspects of the combined feature set by computing attention scores across various dimensions. Following the attention mechanism, residual connections and layer normalization are applied to maintain gradient flow. The output is further processed by a fully connected feed-forward network consisting of two linear transformations separated by a ReLU activation function. Through this architecture, the Transformer is capable of learning complex dependencies between the preoperative and postoperative features, which is essential for making accurate tumor response predictions. The final output of the Transformer is passed through a softmax layer for classification.

1. **Training**

During the model’s training phase, for each patient, we selected the CE-MRI slice with the largest tumor cross-sectional area as input. These ROI slices, covering the entirety of the tumor, were meticulously annotated and used as independent inputs in the training process. During training, for each patient, we selected the CE-MRI slice with the largest tumor cross-sectional area as input. These ROI slices were independently used as inputs to train the deep learning models. A batch size of 2048, 1000 epochs, a learning rate of 0.01, and the AdamW optimizer were used to train the Transformer, whereas the DL_Pre and DL_Post models were trained with 100 epochs using the Adam optimizer (learning rates: 0.0001 and 0.001; batch sizes: 256 and 64, respectively). To prevent overfitting, L2 regularization and early stopping were applied during training. The models’ performance was evaluated by minimizing a loss function, which was a key focus throughout the training process. Data augmentation, widely used to enhance the quantity and diversity of training samples, was applied to improve the generalization of the models. Specifically, the data augmentation was designed to address variations in image intensity across different MRI protocols. Techniques such as horizontal and vertical flipping, as well as random cropping, were employed to further diversify the training data. These augmentations were implemented using the Pytorch, with augmentation applied dynamically during the generation of training batches.

1. **Loss function**

The softmax cross-entropy loss function was chosen for model training, as it is well-suited for classification tasks. The softmax function converts the model’s output into a probability distribution, enabling the comparison of predicted and actual class distributions. Cross-entropy measures the divergence between these distributions, penalizing the model for incorrect or underconfident predictions. This makes the loss function particularly useful in cases of class imbalance or where confidence in predictions is critical. By minimizing the softmax cross-entropy loss, the model learns to produce confident, accurate classifications of tumor response, while maintaining robustness across different response categories.

Full implementation details, including model architecture, feature extraction pipeline, training code, and reproducibility instructions, are available at the following GitHub repository: [https://github.com/OnekeyAI-Platform/onekey](https://github.com/OnekeyAI-Platform/onekey).

This repository provides access to source code and additional documentation supporting the methodological framework described in this study.

**S8. Feature Selection Algorithm**

1. **ICC (Intraclass correlation coefficient)**

A statistical measure used to assess the reliability and agreement between different raters or measurements. It evaluates how consistently radiologists segment the same ROI, ensuring that extracted features are robust and not overly influenced by the variability between raters. To assess feature reproducibility, a subset of 20 patients (training vs validation cohort =15 : 5) was randomly selected for interobserver analysis. This random sampling approach was used to ensure representativeness across the dataset and to avoid selection bias in evaluating segmentation consistency.

1. **Mann-Whitney U test and t-test**

the appropriate statistical test was selected based on whether the data followed a normal distribution. For features that followed a normal distribution, the t-test (Student's t-test) was used to compare differences between the two groups (OR and NR groups). For features that did not follow a normal distribution, the non-parametric U-test (Mann-Whitney U test) was employed. Both tests were used to assess whether the distributions of features differed significantly between the groups. Features with *p* values < 0.05 were considered statistically significant and retained.

1. **Spearman Correlation**

A statistical method employed to assess the strength and direction of a monotonic relationship between two variables. In the context of feature selection, it is used to identify multicollinearity between features. Highly correlated features (≥ 0.9 or ≤ −0.9) indicate redundancy, and only one feature from each pair, typically the one with better diagnostic performance, is retained.

1. **MRMR (Minimum Redundancy Maximum Relevance)**

A feature selection technique that aims to select features that are highly relevant to the outcome variable while minimizing redundancy among them. MRMR is particularly effective when dealing with high-dimensional data, as it selects a subset of features that are both informative and non-redundant, retaining up to 20 features to improve the model's performance.

1. **LASSO (least absolute shrinkage and selection operator)**

Lasso regression is a linear model that estimates sparse coefficients, favoring solutions with fewer non-zero coefficients, which reduces the number of dependent features in the model. This makes Lasso particularly valuable in certain applications, as it serves as a foundational method in the field of compressed sensing. Under specific conditions, Lasso can accurately identify the set of non-zero coefficients. Due to its ability to produce sparse models, Lasso is commonly used for feature selection, especially in the context of L1-based feature selection methods.

1. **PCA (Principal Component Analysis)**

A dimensionality reduction technique used to transform high-dimensional data into a smaller set of uncorrelated components, called principal components. By capturing the most variance in the data with fewer dimensions, PCA reduces the computational complexity and enhances the performance of subsequent feature selection methods. In this study, PCA was applied specifically to the deep learning features extracted by ResNet50.


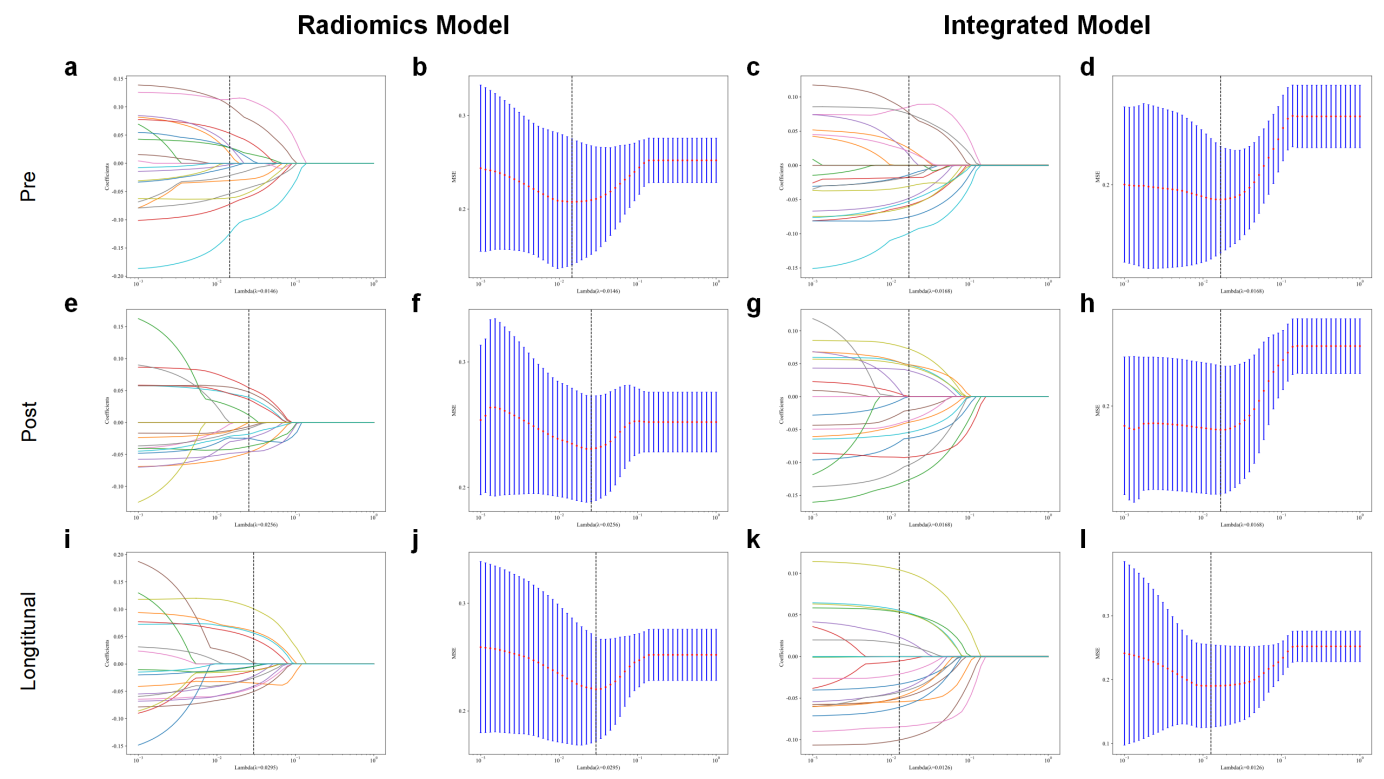


**Figure S2. The least absolute shrinkage and selection operator (LASSO) features selection in training cohort in the six models. LASSO feature selection results for radiomics and integrated models using different time points images.(a-d) Pre-treatment models, (e-h) post-treatment models, and (i-l) longitudinal models. Panels on the left (a, e, i, c, g, k) show the LASSO coefficient profiles as a function of the regularization parameter lambda, with each colored line representing one feature. The vertical dashed line indicates the optimal lambda selected via 5-fold cross-validation. Panels on the right (b, f, j, d, h, l) show the mean squared error (MSE) and standard deviation for each lambda value during 5-cross-validation. The lambda value with the minimum MSE (red dots) or within one standard error is chosen to balance model performance and complexity.**

**S9. Machine Learning Algorithms**

1. **Random Forest**

Random Forest is an ensemble learning method that consists of multiple decision trees, used for both classification and regression problems, and capable of handling multi-class problems. It is a non-linear model that predicts by sending a test sample to each decision tree for classification, and the final output is determined by majority voting among the trees. Instead of selecting the best split at each node among all features, it randomly selects the best split from a subset of features, increasing the model’s robustness and reducing overfitting. Hyperparameters such as “n_estimators” (number of trees) and “criterion” (splitting measure) were tuned during model development.

1. **eXtreme Gradient Boosting (XGboost)**

eXtreme Gradient Boosting is a scalable, tree-based algorithm that builds decision trees sequentially. Each subsequent tree corrects errors from the previous trees by minimizing a gradient descent loss function. It supports both classification and regression tasks. Important tuning parameters include “learning_rate” (step size shrinkage) and “n_estimators”.

1. **Support Vector Machine (SVM)**

SVM is a supervised learning algorithm that excels in classification tasks, particularly in high-dimensional, small-sample datasets. It finds the optimal hyperplane that maximizes the margin between different classes. SVM supports both linear and non-linear classification by using kernel functions to project data into higher-dimensional spaces. The key hyperparameters include “C” (regularization parameter) and “gamma” (kernel coefficient).

1. **K-Nearest Neighbors (KNN)**

KNN is a simple, instance-based learning algorithm that classifies a test sample based on the majority class among its “k” nearest neighbors in the feature space. It is a non-parametric model that does not make assumptions about the underlying data distribution. The number of neighbors “k” and the distance metric (such as Euclidean distance) are critical hyperparameters.

1. **Light Gradient Boosting Machine (LightGBM)**

LightGBM is an efficient implementation of gradient boosting that uses decision tree learning. It differs from other gradient boosting algorithms by using leaf-wise tree growth, which can reduce overfitting and improve model accuracy. LightGBM supports both classification and regression tasks. Important tuning parameters include “num_leaves” (number of leaves in a tree) and “learning_rate”.

1. **Extremely Randomized Trees (ExtraTrees)**

Extremely Randomized Trees is an ensemble learning method that operates by constructing multiple decision trees, similar to Random Forest. However, ExtraTrees introduces more randomness by selecting random split points at each node. This additional randomness helps prevent overfitting while maintaining model accuracy. Important hyperparameters include “n_estimators” (number of trees) and “max_features” (number of features to consider for each split).

**S10. Shapley Additive exPlanations(SHAP) Algorithms**

SHAP is a unified framework that provides insights into machine learning model predictions. Based on principles from cooperative game theory, SHAP quantifies the contribution of individual features to the model’s predictions by calculating Shapley values. Each Shapley value represents the marginal contribution of a feature by comparing model outputs with and without that specific feature, averaged over all possible combinations of features. This method is particularly powerful in addressing the “black-box” nature of models such as SVM by offering both global and local interpretability.

Given the nature of our SVM model, we employed the SHAP kernel explainer, a model-agnostic method that can approximate Shapley values for any machine learning model. The kernel explainer models each patient’s features as “players” in a cooperative game, and the model's prediction as the “payout” from the game. The Shapley values, computed by the kernel explainer, represent how much each feature contributes to moving the prediction away from a baseline value (the mean prediction across the dataset). For each sample, the kernel explainer considers multiple combinations of the input features to compute the marginal contribution of each feature. This approach is useful for SVM models, which often operate as black boxes and lack inherent interpretability. By computing the SHAP values, we are able to assign importance scores to individual features and explain the model's decision-making process on a per-patient basis.

Two key types of SHAP visualizations were used in our study to provide both global and local explanations:

1. SHAP beewarm Plot (global):

The SHAP beewarm plot provides an overview of feature importance across the dataset. Each dot on the plot represents a single patient, and the color of the dot indicates the feature value (high or low). The horizontal axis represents the SHAP value, which shows the magnitude and direction (positive or negative) of the feature's influence on the prediction. This plot allowed us to visualize which features were consistently important across all patients and how they impacted the model’s output. In our final SVM model, Post_DL_1 emerged as the most globally influential feature (Figure 4a).

1. SHAP Force Plot (local):

SHAP force plots were used to explain individual predictions. These visualizations show how each feature either pushes the prediction higher or pulls it lower compared to the baseline value (the average prediction for the dataset). The force plot provides a clear and intuitive representation of how features interact to generate the final prediction for a single patient, making it particularly useful for understanding complex individual cases. In figure 4b and 4c, Post_DL_1 showed the strongest impact on the model’s decision at the individual level.

1. SHAP waterfall Plot (local):

The SHAP waterfall plot provides a step-by-step breakdown of the contribution of each feature in driving the prediction away from the baseline. Starting from the baseline value (the average prediction across all patients), each feature incrementally adds or subtracts from this baseline until reaching the model’s final prediction for an individual case. The visualization arranges features in order of their importance, showing the most influential features at the top. Each “step” in the waterfall plot corresponds to a feature’s SHAP value, with positive contributions pushing the prediction up and negative contributions pulling it down. This visual layout makes it easy to see not only which features are most impactful but also whether they contribute to an increase or decrease in the prediction. In our study, the SHAP waterfall plot was instrumental in identifying the main drivers of specific predictions, allowing us to investigate the most significant factors influencing individual patient outcomes within the SVM model's predictions. In our final model, Post_DL_1 was consistently the most impactful feature in determining prediction direction and strength.

**S11. Features selection results**

For the Radiomics Features Only Model, the feature selection for Rad_Pre, Rad_Post, and Rad_Delta resulted in the identification of 15, 13, and 15 final features, respectively.

1. Rad_Pre included 2 first-order features, 1 shape-based feature, 4 texture features, 8 wavelet features;
2. Rad_Post included 3 first-order features, 1 shape-based feature, 9 wavelet features;
3. Rad_Delta included 3 first-order features, 4 texture features, 8 wavelet features.

For the Integrated Model, combining DRF and SDF, feature selection for DL_Rad_Pre, DL_Rad_Post, and DL_Rad_Delta yielded 16, 14, and 17 final features, respectively.

1. DL_Rad_Pre with 2 first-order features, 1 shape-based feature, 5 texture features, 5 wavelet features, and 3 DLF;
2. DL_Rad_Post with 2 first-order features, texture features, 5 wavelet features, and 7 DLF;
3. DL_Rad_Delta with 3 first-order features, 3 texture features, 7 wavelet features, and 4 DLF.

Feature name of the DL_Rad_Delta Model:

1. Pre_T1WI_Portal_wavelet_LHH_glszm_SmallAreaEmphasis
2. Post_T1WI_Delay_log_sigma_3_0_mm_3D_firstorder_Kurtosis
3. Pre_T1WI_Portal_log_sigma_4_0_mm_3D_glszm_LargeAreaLowGrayLevelEmphasis
4. Pre_T1WI_Portal_log_sigma_4_0_mm_3D_glcm_ClusterShade
5. Post_T1WI_Portal_wavelet_LHH_glcm_ClusterShade
6. Pre_T1WI_Arterial_log_sigma_2_0_mm_3D_firstorder_Kurtosis
7. Post_T1WI_Portal_original_firstorder_Kurtosis
8. Pre_T1WI_Delay_log_sigma_5_0_mm_3D_glrlm_GrayLevelVariance
9. Post_T1WI_Arterial_wavelet_LLH_glszm_SmallAreaLowGrayLevelEmphasis
10. Pre_DL_18
11. Post_T1WI_Arterial_wavelet_HHH_firstorder_Kurtosis
12. Pre_T1WI_Portal_wavelet_HHH_glcm_InverseVariance
13. Post_T1WI_Delay_wavelet_LLH_firstorder_Kurtosis
14. Post_DL_4
15. Post_DL_1
16. Post_T1WI_Portal_wavelet_HLL_firstorder_Median
17. Post_DL_41

**S12. Delong test results**

**
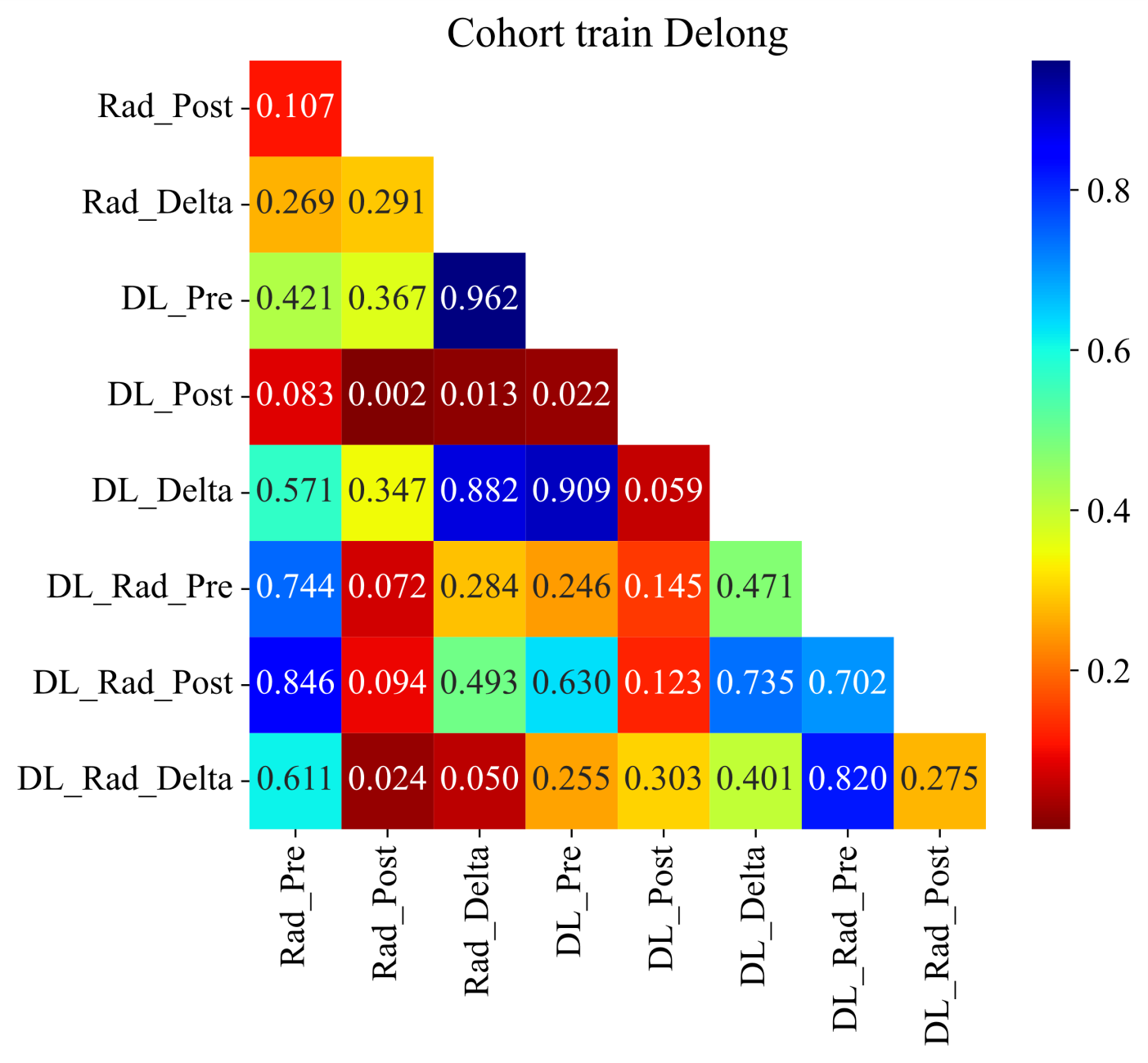
**

**Figure S3. AUCs of all models for tumor response prediction in training cohort, in HCC patients with DEB-TACE. *p* value refers to Delong test for the differences of AUCs between different metrics in different models; *p* < 0.05, with significant differences for AUCs.**

**
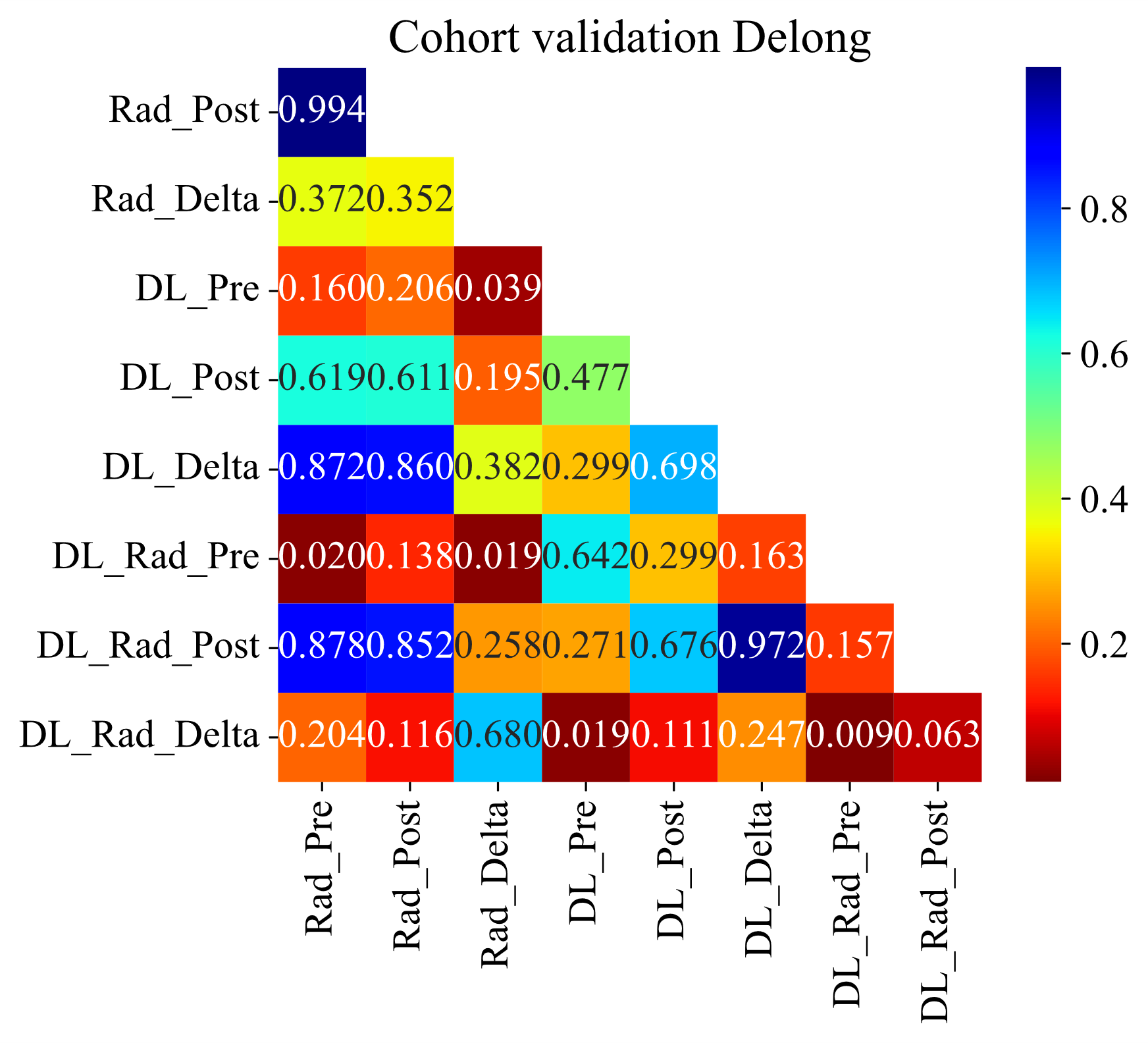
**

**Figure S4. AUCs of all models for tumor response prediction in validation cohort, in HCC patients with DEB-TACE. *p* value refers to Delong test for the differences of AUCs between different metrics in different models; *p* < 0.05, with significant differences for AUCs.**

**S13. Model performance**

**Table S3. The performance of different machine learning models using Radiomics Features from Pre CE-MRI**

|  | Model_name | Accuracy | AUC | 95%CI | Sensitivity | Specificity | PPV | NPV | Precision | Recall | F1 | Threshold | Cohort |
| --- | --- | --- | --- | --- | --- | --- | --- | --- | --- | --- | --- | --- | --- |
| Rad  Pre | **SVM** | **0.844** | **0.928** | **0.8861-0.9692** | **0.875** | **0.803** | **0.854** | **0.831** | **0.854** | **0.875** | **0.864** | **0.533** | **training** |
|  | **SVM** | **0.754** | **0.848** | **0.7456-0.9508** | **0.698** | **0.889** | **0.937** | **0.552** | **0.937** | **0.698** | **0.800** | **0.585** | **validation** |
|  | KNN | 0.596 | 0.864 | 0.8079-0.9206 | 0.287 | 1.000 | 1.000 | 0.517 | 1.000 | 0.287 | 0.447 | 0.800 | training |
|  | KNN | 0.410 | 0.736 | 0.6128-0.8601 | 0.163 | 1.000 | 1.000 | 0.333 | 1.000 | 0.163 | 0.280 | 0.800 | validation |
|  | RandomForest | 0.879 | 0.937 | 0.8956-0.9775 | 0.837 | 0.934 | 0.944 | 0.814 | 0.944 | 0.837 | 0.887 | 0.543 | training |
|  | RandomForest | 0.705 | 0.667 | 0.5076-0.8270 | 0.791 | 0.500 | 0.791 | 0.500 | 0.791 | 0.791 | 0.791 | 0.458 | validation |
|  | ExtraTrees | 0.759 | 0.850 | 0.7883-0.9117 | 0.812 | 0.689 | 0.774 | 0.737 | 0.774 | 0.812 | 0.793 | 0.563 | training |
|  | ExtraTrees | 0.770 | 0.682 | 0.5296-0.8334 | 0.930 | 0.389 | 0.784 | 0.700 | 0.784 | 0.930 | 0.851 | 0.477 | validation |
|  | XGBoost | 0.943 | 0.982 | 0.9638-1.0000 | 0.937 | 0.951 | 0.962 | 0.921 | 0.962 | 0.937 | 0.949 | 0.565 | training |
|  | XGBoost | 0.672 | 0.651 | 0.4929-0.8081 | 0.721 | 0.556 | 0.795 | 0.455 | 0.795 | 0.721 | 0.756 | 0.483 | validation |
|  | LightGBM | 0.865 | 0.909 | 0.8563-0.9613 | 0.862 | 0.869 | 0.896 | 0.828 | 0.896 | 0.862 | 0.879 | 0.562 | training |
|  | LightGBM | 0.541 | 0.660 | 0.5101-0.8090 | 0.419 | 0.833 | 0.857 | 0.375 | 0.857 | 0.419 | 0.562 | 0.601 | validation |

PPV, positive pretictive value; NPV, negative pretictive value; CI, confidence interval; AUC, area under the curve; SVM, support vector machine; KNN, K nearest neighbors; XGBoost, extreme gradient boosting; LightGBM, light gradient boosting machine

**Table S4. The performance of different machine learning models using Radiomics Features from Post CE-MRI**

|  | Model_name | Accuracy | AUC | 95%CI | Sensitivity | Specificity | PPV | NPV | Precision | Recall | F1 | Threshold | Cohort |
| --- | --- | --- | --- | --- | --- | --- | --- | --- | --- | --- | --- | --- | --- |
| Rad  Post | **SVM** | **0.780** | **0.871** | **0.8130-0.9292** | **0.762** | **0.803** | **0.836** | **0.721** | **0.836** | **0.762** | **0.797** | **0.577** | **training** |
|  | **SVM** | **0.836** | **0.849** | **0.7357-0.9620** | **0.907** | **0.667** | **0.867** | **0.750** | **0.867** | **0.907** | **0.886** | **0.508** | **validation** |
|  | KNN | 0.660 | 0.815 | 0.7479-0.8816 | 0.450 | 0.934 | 0.900 | 0.564 | 0.900 | 0.450 | 0.600 | 0.600 | training |
|  | KNN | 0.361 | 0.631 | 0.4891-0.7732 | 0.116 | 0.944 | 0.833 | 0.309 | 0.833 | 0.116 | 0.204 | 0.800 | validation |
|  | RandomForest | 0.823 | 0.888 | 0.8332-0.9420 | 0.950 | 0.656 | 0.784 | 0.909 | 0.784 | 0.950 | 0.859 | 0.496 | training |
|  | RandomForest | 0.770 | 0.745 | 0.6058-0.8851 | 0.837 | 0.611 | 0.837 | 0.611 | 0.837 | 0.837 | 0.837 | 0.474 | validation |
|  | ExtraTrees | 0.738 | 0.804 | 0.7318-0.8766 | 0.800 | 0.656 | 0.753 | 0.714 | 0.753 | 0.800 | 0.776 | 0.559 | training |
|  | ExtraTrees | 0.754 | 0.826 | 0.7005-0.9520 | 0.698 | 0.889 | 0.937 | 0.552 | 0.937 | 0.698 | 0.800 | 0.571 | validation |
|  | XGBoost | 0.929 | 0.974 | 0.9517-0.9966 | 0.950 | 0.902 | 0.927 | 0.932 | 0.927 | 0.950 | 0.938 | 0.533 | training |
|  | XGBoost | 0.803 | 0.824 | 0.7075-0.9411 | 0.837 | 0.722 | 0.878 | 0.650 | 0.878 | 0.837 | 0.857 | 0.456 | validation |
|  | LightGBM | 0.801 | 0.873 | 0.8140-0.9311 | 0.787 | 0.820 | 0.851 | 0.746 | 0.851 | 0.787 | 0.818 | 0.562 | training |
|  | LightGBM | 0.787 | 0.836 | 0.7249-0.9469 | 0.767 | 0.833 | 0.917 | 0.600 | 0.917 | 0.767 | 0.835 | 0.524 | validation |

PPV, positive pretictive value; NPV, negative pretictive value; CI, confidence interval; AUC, area under the curve; SVM, support vector machine; KNN, K nearest neighbors; XGBoost, extreme gradient boosting; LightGBM, light gradient boosting machine

**Table S5. The performance of different machine learning models using Delta Radiomics Features from longitudinal Pre CE-MRI**

|  | Model_name | Accuracy | AUC | 95%CI | Sensitivity | Specificity | PPV | NPV | Precision | Recall | F1 | Threshold | Cohort |
| --- | --- | --- | --- | --- | --- | --- | --- | --- | --- | --- | --- | --- | --- |
| Rad Delta | **SVM** | **0.837** | **0.903** | **0.8510-0.9543** | **0.837** | **0.836** | **0.870** | **0.797** | **0.870** | **0.837** | **0.854** | **0.551** | **training** |
|  | **SVM** | **0.885** | **0.910** | **0.8199-0.9993** | **0.907** | **0.833** | **0.929** | **0.789** | **0.929** | **0.907** | **0.918** | **0.453** | **validation** |
|  | KNN | 0.582 | 0.867 | 0.8119-0.9223 | 0.262 | 1.000 | 1.000 | 0.508 | 1.000 | 0.262 | 0.416 | 0.800 | training |
|  | KNN | 0.541 | 0.726 | 0.5963-0.8559 | 0.395 | 0.889 | 0.895 | 0.381 | 0.895 | 0.395 | 0.548 | 0.600 | validation |
|  | RandomForest | 0.816 | 0.892 | 0.8378-0.9456 | 0.750 | 0.902 | 0.909 | 0.733 | 0.909 | 0.750 | 0.822 | 0.616 | training |
|  | RandomForest | 0.770 | 0.830 | 0.6970-0.9632 | 0.721 | 0.889 | 0.939 | 0.571 | 0.939 | 0.721 | 0.816 | 0.565 | validation |
|  | ExtraTrees | 0.780 | 0.826 | 0.7554-0.8964 | 0.900 | 0.623 | 0.758 | 0.826 | 0.758 | 0.900 | 0.823 | 0.572 | training |
|  | ExtraTrees | 0.721 | 0.799 | 0.6668-0.9314 | 0.698 | 0.778 | 0.882 | 0.519 | 0.882 | 0.698 | 0.779 | 0.583 | validation |
|  | XGBoost | 0.965 | 0.990 | 0.9784-1.0000 | 0.950 | 0.984 | 0.987 | 0.937 | 0.987 | 0.950 | 0.968 | 0.606 | training |
|  | XGBoost | 0.639 | 0.715 | 0.5864-0.8438 | 0.535 | 0.889 | 0.920 | 0.444 | 0.920 | 0.535 | 0.676 | 0.658 | validation |
|  | LightGBM | 0.865 | 0.930 | 0.8889-0.9713 | 0.950 | 0.754 | 0.835 | 0.920 | 0.835 | 0.950 | 0.889 | 0.505 | training |
|  | LightGBM | 0.770 | 0.795 | 0.6749-0.9155 | 0.791 | 0.722 | 0.872 | 0.591 | 0.872 | 0.791 | 0.829 | 0.499 | validation |

PPV, positive pretictive value; NPV, negative pretictive value; CI, confidence interval; AUC, area under the curve; SVM, support vector machine; KNN, K nearest neighbors; XGBoost, extreme gradient boosting; LightGBM, light gradient boosting machine

**Table S6. The performance of different machine learning models using Deep Learning Features and Radiomics Features from Pre CE-MRI**

|  | Model_name | Accuracy | AUC | 95%CI | Sensitivity | Specificity | PPV | NPV | Precision | Recall | F1 | Threshold | Cohort |
| --- | --- | --- | --- | --- | --- | --- | --- | --- | --- | --- | --- | --- | --- |
| Integrated Pre | **SVM** | **0.865** | **0.934** | **0.8942-0.9745** | **0.887** | **0.836** | **0.877** | **0.850** | **0.877** | **0.887** | **0.882** | **0.482** | **training** |
|  | **SVM** | **0.672** | **0.695** | **0.5393-0.8509** | **0.628** | **0.778** | **0.871** | **0.467** | **0.871** | **0.628** | **0.730** | **0.563** | **validation** |
|  | KNN | 0.759 | 0.887 | 0.8375-0.9373 | 0.625 | 0.934 | 0.926 | 0.655 | 0.926 | 0.625 | 0.746 | 0.600 | training |
|  | KNN | 0.590 | 0.685 | 0.5330-0.8378 | 0.535 | 0.722 | 0.821 | 0.394 | 0.821 | 0.535 | 0.648 | 0.600 | validation |
|  | RandomForest | 0.915 | 0.949 | 0.9101-0.9887 | 0.925 | 0.902 | 0.925 | 0.902 | 0.925 | 0.925 | 0.925 | 0.541 | training |
|  | RandomForest | 0.393 | 0.504 | 0.3375-0.6703 | 0.163 | 0.944 | 0.875 | 0.321 | 0.875 | 0.163 | 0.275 | 0.758 | validation |
|  | ExtraTrees | 0.830 | 0.927 | 0.8869-0.9664 | 0.712 | 0.984 | 0.983 | 0.723 | 0.983 | 0.712 | 0.826 | 0.599 | training |
|  | ExtraTrees | 0.689 | 0.645 | 0.4863-0.8032 | 0.721 | 0.611 | 0.816 | 0.478 | 0.816 | 0.721 | 0.765 | 0.514 | validation |
|  | XGBoost | 0.972 | 0.995 | 0.9884-1.0000 | 0.975 | 0.967 | 0.975 | 0.967 | 0.975 | 0.975 | 0.975 | 0.549 | training |
|  | XGBoost | 0.639 | 0.554 | 0.3820-0.7266 | 0.698 | 0.500 | 0.769 | 0.409 | 0.769 | 0.698 | 0.732 | 0.478 | validation |
|  | LightGBM | 0.858 | 0.905 | 0.8563-0.9540 | 0.925 | 0.770 | 0.841 | 0.887 | 0.841 | 0.925 | 0.881 | 0.521 | training |
|  | LightGBM | 0.607 | 0.587 | 0.4274-0.7470 | 0.605 | 0.611 | 0.788 | 0.393 | 0.788 | 0.605 | 0.684 | 0.538 | validation |

PPV, positive pretictive value; NPV, negative pretictive value; CI, confidence interval; AUC, area under the curve; SVM, support vector machine; KNN, K nearest neighbors; XGBoost, extreme gradient boosting; LightGBM, light gradient boosting machine

**Table S7. The performance of different machine learning models using Deep Learning Features and Radiomics Features from Post CE-MRI**

|  | Model_name | Accuracy | AUC | 95%CI | Sensitivity | Specificity | PPV | NPV | Precision | Recal | F1 | Threshold | Cohort |
| --- | --- | --- | --- | --- | --- | --- | --- | --- | --- | --- | --- | --- | --- |
| Integrated Post | **SVM** | **0.887** | **0.921** | **0.8679-0.9747** | **0.925** | **0.836** | **0.881** | **0.895** | **0.881** | **0.925** | **0.902** | **0.581** | **training** |
|  | **SVM** | **0.689** | **0.837** | **0.7385-0.9360** | **0.558** | **1.000** | **1.000** | **0.486** | **1.000** | **0.558** | **0.716** | **0.650** | **validation** |
|  | KNN | 0.667 | 0.808 | 0.7399-0.8760 | 0.537 | 0.836 | 0.811 | 0.580 | 0.811 | 0.537 | 0.647 | 0.600 | training |
|  | KNN | 0.508 | 0.749 | 0.6049-0.8938 | 0.372 | 0.833 | 0.842 | 0.357 | 0.842 | 0.372 | 0.516 | 0.600 | validation |
|  | RandomForest | 0.872 | 0.930 | 0.8873-0.9721 | 0.900 | 0.836 | 0.878 | 0.864 | 0.878 | 0.900 | 0.889 | 0.519 | training |
|  | RandomForest | 0.803 | 0.841 | 0.7310-0.9511 | 0.791 | 0.833 | 0.919 | 0.625 | 0.919 | 0.791 | 0.850 | 0.551 | validation |
|  | ExtraTrees | 0.872 | 0.930 | 0.8878-0.9712 | 0.887 | 0.852 | 0.887 | 0.852 | 0.887 | 0.887 | 0.887 | 0.561 | training |
|  | ExtraTrees | 0.738 | 0.739 | 0.5828-0.8953 | 0.744 | 0.722 | 0.865 | 0.542 | 0.865 | 0.744 | 0.800 | 0.565 | validation |
|  | XGBoost | 0.957 | 0.993 | 0.9851-1.0000 | 0.962 | 0.951 | 0.962 | 0.951 | 0.962 | 0.962 | 0.962 | 0.553 | training |
|  | XGBoost | 0.738 | 0.798 | 0.6670-0.9286 | 0.721 | 0.778 | 0.886 | 0.538 | 0.886 | 0.721 | 0.795 | 0.482 | validation |
|  | LightGBM | 0.894 | 0.945 | 0.9078-0.9830 | 0.900 | 0.885 | 0.911 | 0.871 | 0.911 | 0.900 | 0.906 | 0.532 | training |
|  | LightGBM | 0.770 | 0.839 | 0.7184-0.9599 | 0.744 | 0.833 | 0.914 | 0.577 | 0.914 | 0.744 | 0.821 | 0.520 | validation |

PPV, positive pretictive value; NPV, negative pretictive value; CI, confidence interval; AUC, area under the curve; SVM, support vector machine; KNN, K nearest neighbors; XGBoost, extreme gradient boosting; LightGBM, light gradient boosting machine

**Table S8. The performance of different machine learning models using Siamese Delta Features and Delta Radiomics Features from longitudinal CE-MRI**

|  | Model_name | Accuracy | AUC | 95%CI | Sensitivity | Specificity | PPV | NPV | Precision | Recall | F1 | Threshold | Cohort |
| --- | --- | --- | --- | --- | --- | --- | --- | --- | --- | --- | --- | --- | --- |
| Integrated Delta | **SVM** | **0.908** | **0.941** | **0.8926-0.9893** | **0.937** | **0.869** | **0.904** | **0.914** | **0.904** | **0.937** | **0.920** | **0.593** | **training** |
|  | **SVM** | **0.869** | **0.925** | **0.8520-0.9982** | **0.837** | **0.944** | **0.973** | **0.708** | **0.973** | **0.837** | **0.900** | **0.439** | **validation** |
|  | KNN | 0.766 | 0.891 | 0.8408-0.9403 | 0.637 | 0.934 | 0.927 | 0.663 | 0.927 | 0.637 | 0.756 | 0.600 | training |
|  | KNN | 0.639 | 0.861 | 0.7622-0.9600 | 0.512 | 0.944 | 0.957 | 0.447 | 0.957 | 0.512 | 0.667 | 0.600 | validation |
|  | RandomForest | 0.851 | 0.929 | 0.8897-0.9683 | 0.937 | 0.738 | 0.824 | 0.900 | 0.824 | 0.937 | 0.877 | 0.492 | training |
|  | RandomForest | 0.770 | 0.855 | 0.7577-0.9529 | 0.721 | 0.889 | 0.939 | 0.571 | 0.939 | 0.721 | 0.816 | 0.549 | validation |
|  | ExtraTrees | 0.823 | 0.877 | 0.8177-0.9368 | 0.850 | 0.787 | 0.840 | 0.800 | 0.840 | 0.850 | 0.845 | 0.575 | training |
|  | ExtraTrees | 0.623 | 0.671 | 0.5134-0.8277 | 0.558 | 0.778 | 0.857 | 0.424 | 0.857 | 0.558 | 0.676 | 0.601 | validation |
|  | XGBoost | 0.972 | 0.996 | 0.9910-1.0000 | 0.987 | 0.951 | 0.963 | 0.983 | 0.963 | 0.987 | 0.975 | 0.596 | training |
|  | XGBoost | 0.607 | 0.743 | 0.6150-0.8708 | 0.442 | 1.000 | 1.000 | 0.429 | 1.000 | 0.442 | 0.613 | 0.680 | validation |
|  | LightGBM | 0.879 | 0.942 | 0.9079-0.9769 | 0.937 | 0.803 | 0.862 | 0.907 | 0.862 | 0.937 | 0.898 | 0.538 | training |
|  | LightGBM | 0.787 | 0.791 | 0.6568-0.9259 | 0.837 | 0.667 | 0.857 | 0.632 | 0.857 | 0.837 | 0.847 | 0.463 | validation |

PPV, positive pretictive value; NPV, negative pretictive value; CI, confidence interval; AUC, area under the curve; SVM, support vector machine; KNN, K nearest neighbors; XGBoost, extreme gradient boosting; LightGBM, light gradient boosting machine
